# Clinicopathological significance of the EMT-related proteins and their interrelationships in prostate cancer. An immunohistochemical study

**DOI:** 10.1371/journal.pone.0253112

**Published:** 2021-06-22

**Authors:** Martyna Parol-Kulczyk, Arkadiusz Gzil, Joanna Maciejewska, Magdalena Bodnar, Dariusz Grzanka

**Affiliations:** Department of Clinical Pathomorphology, Faculty of Medicine, Collegium Medicum in Bydgoszcz, Nicolaus Copernicus University in Torun, Toruń, Poland; University of Nebraska Medical Center, UNITED STATES

## Abstract

The chronic inflammation influences a microenvironment, where as a result of losing control over tissue homeostatic mechanisms, the carcinogenesis process may be induced. Inflammatory response cells can secrete a number of factors that support both initiation and progression of cancer and also they may consequently induct an epithelial-mesenchymal transition (EMT), the process responsible for development of distant metastasis. Macrophage migration inhibitory factor (MIF) acts as a pro-inflammatory cytokine that is considered as a link between chronic inflammation and tumor development. MIF can function as a modulator of important cancer-related genes expression, as well as an activator of signaling pathways that promotes the development of prostate cancer. The study was performed on FFPE tissues resected from patients who underwent radical prostatectomy. To investigate the relationship of studied proteins with involvement in tumor progression and initiation of epithelial-to-mesenchymal transition (EMT) process, we selected clinicopathological parameters related to tumor progression. Immunohistochemical analyses of MIF, SOX-4, β-catenin and E-cadherin were performed on TMA slides. We found a statistically significant correlation of overall β-catenin expression with the both lymph node metastasis (p<0.001) and presence of angioinvasion (p = 0.012). Membrane β-catenin expression was associated with distant metastasis (p = 0.021). In turn, nuclear MIF was correlated with lymph node metastasis (p = 0.003). The positive protein-protein correlations have been shown between the total β-catenin protein expression level with level of nuclear SOX-4 protein expression (r = 0.27; p<0.05) as well as negative correlation of β-catenin expression with level of nuclear MIF protein expression (r = -0.23; p<0.05). Our results seem promising and strongly highlight the potential role of MIF in development of nodal metastases as well as may confirm an involvement of β-catenin in disease spread in case of prostate cancer.

## Introduction

Prostate cancer (PCa) is the most common malignancy as well as the fifth cause of cancer-related death among men worldwide [1]. In 2020, it was estimated approximately 191,930 of PCa new cases and 33,330 of PCa–related deaths occurred in the United States [2]. The PCa incidence rate varies depending on genetic, hormonal-dependent and environmental aspects. Besides, the PCa prevalence and mortality rate are associated with elderly age and the average age at the time of diagnosis is above 65 years [1].

PCa still remains a highly treatable neoplasm if it is diagnosed as localized disease at its an early stage and constantly monitored [3]. These patients could be usually treated by radical prostatectomy or radiotherapy, which guarantee a successful treatment outcome in most cases. However, the National Cancer Institute indicated that the lymph node metastasis are observed in 12% of PCa patients at the time of diagnosis [4]. Additionally, by the significant proportion of patients the PCa undergo progression to a highly advanced, metastatic stage of disease for which treatment options are limited and the prognosis is uncertain [5]. Based on the Surveillance, Epidemiology, and End Results Program (SEER) database, the 5-year relative survival rate for PCa cases with localized and regional stage is at 100%, whereas at the metastatic stage the level is only 29% [6].

Solid tumors, such as PCa grow in hypoxic conditions, which are characterized by inadequate blood flow and impaired tumor vessels [7]. These conditions and mutations in the *CTNNB1* gene may lead to activation, accumulation and nuclear translocation of β-catenin [8]. In the consequence there are activated or deactivated specific targets such as transcription factors, cell-surface and cytoskeletal proteins, extracellular matrix degradation enzymes (ECM-degrading enzymes) and specific microRNAs, leading to deregulation of cell proliferation management and inhibition of apoptosis [9]. Described process is called an epithelial-mesenchymal transition (EMT), during which cells lose their epithelial features responsible for cell-to-cell adhesion inter alia as a result of losing of epithelial markers and gain mesenchymal properties. Consequently, initiation of the EMT process results in the cell phenotype changes. The downregulation of epithelial markers, in particular E-cadherin, leads to destruction in cell junction proteins and in consequence to loss of the stable epithelial polarized phenotype of the involved cells. Moreover, they gain mesenchymal markers, like N-cadherin, Vimentin or Fibronectin etc. and ability to migration from their origin place, through modulating signal transduction pathways, such as Wnt/β-catenin and TGF-β pathway, as well as EMT transcription factors: zinc finger E-box binding homeobox (Zeb 1/2) and Snail [10, 11]. The cells with mesenchymal phenotype present invasive and metastatic potential as well as they acquire stemness status (ability to self-renew and differentiate) or also chemoresistance properties [12, 13].

Critical point of the EMT constitutes Wnt/β-catenin signal transduction pathway, where β-catenin acts as a central effector of multiple running separately cellular processes [11]. β-catenin forms catenin-cadherin complex due to binding to the major component of cell-cell adherens junctions (AJ), a membrane anchored E-cadherin, thus enabling modulation of localized cytoskeletal remodeling [14–16]. Mechanism of forming catenin-cadherin communication in AJ ensures β-catenin stabilization and protects it from proteasomal degradation [17, 18]. Loss of E-cadherin leads to collapse of cell-cell connections through spontaneous disruption of AJ and to β-catenin detachment from the cell adhesion complex [19]. Free membrane β-catenin released into the cytoplasm may eventually be translocated to the cell nucleus [11, 19]. β-catenin nuclear translocation is driven through Wnt/β-catenin pathway in cooperation with curly receptors and LRP5/6 co-receptors [20], resulting in the repression of glycogen synthase kinase 3β (GSK3β), which is responsible for ubiquitination of β-catenin together with proteasomal degradation through its phosphorylation and β-catenin stabilization [21]. Transport of β-catenin to the cytoplasm area or nucleus can recruit and stimulate downstream transcription factors of TCF4, thus promoting cell proliferation and tumorigenesis [20, 22]. Stabilized β-catenin is accumulated in cell nucleus, where as a cofactor it interacts with the high-mobility-group (HMG) box family of T-cell factor (TCF)/lymphoid enhancer factor (LEF) transcription factors to modulate target genes of the Wnt pathway [23, 24].

Previous studies showed that transcription factor Sry-related HMG-BOX gene 4 (SOX-4) interacts with members of TCF/LEF family via its HMB domain, which regulates stability of these proteins and therefore indirectly also stability of nuclear translocated β-catenin, which is in turn modulated via TCF/LEF complex [25]. Moreover, the study with gain- and loss-of-function have demonstrated that the SOX-4 may be responsible for enhanced β-catenin/TCF activity and the tumor cells proliferation of SW480 colon carcinoma cell lines [25].

Furthermore, the latest study confirmed that SOX-4 constitutes a crucial point in metastatic progression. Under normal physiological conditions, SOX-4 belonging to sex-determining region Y (SRY) box family with special DNA-binding domain (DBD) in HMG [26] functions as a transcription factor, involved in maintenance of pluripotency in stem cells and wide range of developmental processes, such as embryogenesis, development of the central as well as peripheral nervous system, heart, osteoblastic, thymocytes and differentiation of lymphocytes [27–30]. Moreover, SOX-4 is critical in directing cell fate [31]. Recent studies have also reported that SOX-4 is associated with tumorigenesis and shows higher expression in human malignant tumors, such as prostate cancer [32], colorectal cancer [25], breast cancer [33], lung cancer [34], gastric cancer [35]. Nevertheless, in some tumors, like melanoma [36] or bladder cancer [37] SOX-4 can behave as a tumor suppressor, promoting cell cycle arrest and apoptosis [31]. Interestingly, there are some studies confirming the interactions between the canonical Wnt/β-catenin signaling pathways and the both SOX-4 as well as a Macrophage migration inhibitory factor (MIF). Currently, many researchers are focused on the inflammation-induced EMT process in various types of cancers. Previous studies have shown MIF as a potential molecular link between chronic inflammation and cancer [38]. Additionally, in pancreatic ductal adenocarcinoma, MIF was found to prompt the transition from epithelial phenotype to mesenchymal state of cancer cells [39]. However, MIF-induced activation of EMT has not been confirmed in prostate cancer patients.

MIF generally functions as a multipotent cytokine involved in regulation of immune and inflammatory responses as well as in certain pathological situations including atherosclerosis, rheumatoid arthritis, systemic lupus erythematosus, inflammatory bowel disease, psoriasis and diabetes [40–42]. MIF expression was found in both extracellular and intracellular cell area. The extracellularly MIF was found to be involved in cell proliferation, adhesion, invasion and homeostasis control, while intracellularly MIF is associated with c-Jun activation domain binding protein-1 (JAB1), the tumor suppressor protein p53 and thiol protein oxidoreductase (TPOR) [43]. MIF is secreted upon inflammatory and stress stimulation by immune, parenchymal and tumor cells [40]. Furthermore, MIF affects the tumor microenvironment facilitating proliferation and growth through promotion of angiogenesis necessary for maintaining tumor growth [26, 44]. Bando et al., in their cohort study, have examined the upregulation of nuclear MIF in breast [45], whereas Verjans and colleagues have shown that the intracellular MIF is related to beneficial properties, while the extracellular MIF was involved in promoting breast cancer cell-stroma interactions [43]. Aberrant expression of MIF protein was observed also in many other types of cancers, such as colon [46, 47], melanoma [48], glioblastoma [49], lung adenocarcinomas [50], renal cancer [51], urothelial cancer [52], pancreas carcinoid [39], thyroid cancer [53] as well as prostate cancer [54].

Altered subcellular expression of MIF, SOX-4, β-catenin and E-cadherin may significantly contribute to tumor aggressiveness. The associations between aforementioned proteins as well as their clinicopathological significance in prostate cancer remains still not fully understood. This article focuses on the study of relationships between EMT related proteins and their clinicopathological aspect.

## Material and methods

The research was approved by the Bioethical Commission of Collegium Medicum in Bydgoszcz of the Nicolaus Copernicus University in Torun, Poland (decision number: *KB 248/2019*). The eighty five patients of the Department of General and Oncological Urology, Collegium Medicum in Bydgoszcz, Nicolaus Copernicus University in Torun (Poland) with confirmed PCa were enrolled in the current study. The participants underwent radical prostatectomy between January 2017 and December 2019. All attendees have provided the written, signed and dated informed consent form. The patients’ medical records have been completely anonymized to protect the identity of participants, before including to the research.

### Tissue specimens

The present study was concerned on immunohistochemical evaluation of protein levels performed with Formalin-fixed paraffin embedded (FFPE) specimens of eighty five prostate cancers patients. The individual research stages have been conducted at the Department of Clinical Pathomorphology, Collegium Medicum in Bydgoszcz, Nicolaus Copernicus University in Torun, Poland.

Histological evaluation of resected tumors was performed with hematoxylin-eosin- (H&E-) stained slides to confirm diagnosis and choose representative tumor areas containing no less than 80% of tumor cells. All samples were assessed by two pathologists who independently performed histological stage classifications according to the 8^th^ Edition American Joint Committee on Cancer (AJCC) Cancer Staging classification system [55], extending the current research by defining a precise tumor location and Gleason pattern or score of extracapsular tumor extension to show their significance in EMT. The tumor hallmarks noted in prostate cancer areas were submitted to descriptive analysis.

### Patients characteristics

The age of enrolled patients ranged from 52 to 81 and the median was 66 years. According to the pathologic T stage, all patients was in advanced tumor stage following: pT3a was confirmed in 40 individuals, pT3b in 39 and pT4 in 4. At the time of diagnosis, the positive lymph node status was affirmed in 17 of cases, the positive distant metastasis occurred in approximately 9 of patients, whereas the presence of angioinvasion was found in 13 of tumor patients. Most tumors manifested Gleason pattern 3+4 and 4+3 with frequency 36.5% and 23%, respectively. Low proportion constituted specimens with Gleason pattern 5+4, 3+5, 3+3 and 4+4 at 3.5%, 5.9%, 8.2% and 9.4% level, sequentially. Samples with tumor invaded areas showing Gleason score (GS) 7 were 59%, with GS8 were 15.3% and with GS9 were 16.5%. Differently, samples with presence of cancer invasive foci that exceeded the capsule of prostate gland presented GS 8 with primary and secondary score of 4 (40%) and GS6 with primary and secondary score of 3 (28.2%). Detailed clinicopathological features of the patients with PCa that underwent the study are summarized in Table 1.

**Table 1:** Clinicopathological characteristics of identified participants.

Variables			% of cases (N = 85)
Age	≤60 years		1.6
	>60 years		82.4
Prostate weight	≤66 g		70.6
	>66 g		27
	Unk		2.4
Stage pT according to TNM	pT3a		47
	pT3b		45.9
	pT4		4.7
	unk		1.2
Stage N according to TNM	N0		80
	N1		20
Stage M according to TNM	M0		89.4
	M1		10.6
Gleason Score	6		8.2
	7		58.8
	8		15.3
	9		16.5
	unk		1.2
Gleason pattern	3+3		8.2
	3+4		36.5
	3+5		5.9
	4+3		22.4
	4+4		9.4
	4+5		12.9
	5+4		3.5
	unk		1.2
Angioinvasion	Nos		15.3
	neg		83.5
	Unk		1.2
Area of tumor extension	Top of the gland	R	100
		L	100
		R+L	100
	Right area	≤50%	76.5
		>50%	21.2
		unk	2.4
	Left area	≤50%	62.4
		>50%	35.3
		unk	2.4
	Extraprostatic extension	R	95.3
		L	87
		R+L	87
	Seminal vesicle invasion	R	44.7
		L	54.1
		R+L	44.7
Gleason score of extraprostatic extension	6		28.2
	7		10.6
	8		40
	9		9.4
	10		4.7
	unk		7.1
Gleason score of extraprostatic extension	3+3		28.2
	3+4		5.9
	4+3		4.7
	4+4		40
	4+5		8.2
	5+4		1.2
	5+5		4.7
	unk		7.1

unk- unknown; pos- positive; neg- negative; R- right area; L-left area.

### Tissue microarray (TMA) construction and immunohistochemistry (IHC) analysis

TMAs were produced by relocating of earlier marked representative tissue cores, obtained from conventional paraffin block representing particular patient and arranged on a five-cores recipient paraffin block. Reflecting the fact that small cores from PCa with such high morphological heterogeneity might not be representative for the whole affected tumor area, we decided to use core diameters of 5 mm, including one core per patient was extracted from tumor area and one core per patient from tumor-free areas, representing tissue control. The Gleason grading and score assessment were done.

TMA blocks were cut with a rotary microtome (Accu-Cut; Sakura, Torrance, CA, USA) to 4.0 μm thick paraffin sections and mounted onto either special adhesive slides (SuperFrost-Plus, Thermo Scientific) for subsequent analyses The highest quality of microscopic evaluation process was ensure by simultaneously performing of IHC staining the both on TMA sections (encompassing tumor and adjacent healthy tissue) and on a positive control material, recommended by the producer or interactive database The Human Protein Atlas available from https://www.proteinatlas.org [56]. Deparaffinization, rehydration and heat-mediated epitope retrieval steps were done with a pH6 antigen retrieval solution in a PT-Link (Dako). The sections were subsequently incubated with the following primary antibodies;: rabbit monoclonal antibody against β-catenin (clone [E247], ab32572, Abcam, Cambridge, UK), rabbit monoclonal antibody against E-cadherin (clone [EP700Y], ab40772, Abcam, Cambridge, UK), rabbit polyclonal antibody against MIF (HPA003868, Sigma-Aldrich) and rabbit polyclonal antibody against SOX-4 (ab86809, Abcam, Cambridge, UK). The samples were incubated with monoclonal antibodies for 25 minutes in 37°C, whereas the incubation time with polyclonal antibodies needed to be extended to 15 h at 4°C. Characteristics of primary antibodies implicated in the current study was presented in Table 2. Immunohistochemical staining was performed in a Benchmark GX Platform automated slide processing system (Ventana Medical Systems, Tucson, AZ, USA) using the OptiView DAB IHC detection kit (Ventana Medical Systems) according to the manufacturer’s instructions. EnVision FLEX-HRP (Dako) was used to detect the antigen-antibody complexes. Finally, the sections were counterstained with Meyer’s hematoxylin, dehydrated in graded ethanols (80, 90, 96, 99.8%), cleared in series of xylenes (I–IV) and sealed with a Dako Mounting Medium.

**Table 2:** Primary antibody characteristics.

**Antibody**	**Primary antibody dilution**	**Positive control according to antibody data sheet and Human Protein Atlas**	**Cellular localization/expression in prostate cancer tissue**	**Catalog number**
Β-catenin	1:500	colon	Membrane/cytoplasmic/nuclear	ab32572
E-cadherin	1:500	colonic adenocarcinoma	Membrane/cytoplasmic/nuclear	ab40772
MIF	1:500	kidney	Cytoplasmic/nuclear	HPA003868
SOX-4	1:100	testis	Cytoplasmic/nuclear	ab86809

The immunohistochemical staining of MIF and SOX-4 was performed manually, using primary antibody against MIF as well as primary antibody against SOX-4 and independently incubation overnight at 4°C. Additionally, the ZytoChem Plus (HRP) One-Step Polymer anti-Mouse/Rabbit/Rat kit (Zytomed) was used to enhance detection signal of SOX-4/antibody complexes. Proteins localization was visualized using DAB as a chromogen.

### Criterion for positive immunohistochemical staining

The protein levels (β-catenin, E-cadherin, SOX-4 and MIF) were scored by protein localization and intensity of IHC staining in malignant cellsusing light microscope ECLIPSE E800 (Nikon Instruments Europe, Amsterdam, The Netherlands). The evaluation step was provided by two independent, board-certified pathologist. Protein expressions were estimated, following the Remmele and Stegner (1987) immunoreactive score (IRS), which is based on the ratio of expression intensity and percentage of positively expressed cells. The staining intensity was evaluated in 4-point scale (expression: negative-0, weak-1, moderate-2, strong-3) and 5-point scale of percentage of positive tumor cells: 0, 1, 2, 3, 4 corresponded respectively to <10%, 10–50%, 51–80% and >80% of positive cells and giving the maximum result of 12.

### Statistical analysis

All statistical analyses were performed using GraphPad Prism version 8 (GraphPad Software Inc.) and Microsoft Excel 2007. The expression values of analyzed proteins were presented 25^th^ percentile (25p), the median (M) and the 75^th^ percentile (75p). Our data does not fit a specific distribution and therefore we used nonparametric statistical tests. The comparative studies were analyzed statistically using the U Mann-Whitney (in case of two comparable groups) and Kruskal-Wallis test (in case of three or more comparable groups). Spearman test were used to investigate the relationship between protein levels and clinicopathological parameters: age, tumor size, pathological tumor stage, Gleason pattern, total Gleason score, presence of lymph node or distant metastases, presence of angioinvasion or plugs in the vessels as well as invasion depth. The p-values below 0.05 were considered statistically significant.

## Results

### Cellular redistribution of immunohistochemically stained EMT-related proteins in PCa

Immunohistochemical evaluation was provided considering protein subcellular localization and IRS score.

The nuclear expression of β-catenin was exhibited in 61 (71.8%) of specimens, while in 82 (96.4%) specimens were displayed cytoplasmic expression of β-catenin. β-catenin membrane staining was observed in 76 (89.4%) of cases. Membrane and cytoplasmic E-cadherin expression was confirmed in 98.8% (84) of specimens, in turn nuclear level of E-cadherin appeared in 97.6% (83) of cases. Strong membrane expression of β-catenin and E-cadherin have been found in normal glandular cells of adjacent healthy prostate tissues, and was treated as an internal positive control. The positive cytoplasmic expression of MIF was detected in 83 (97.6%) samples, and the positive nuclear MIF expression was assessed in 55 (64.6%) cases, whereas in healthy adjacent prostate tissue the normal glandular and immune cells appeared a strong cytoplasmic staining pattern of MIF protein and it was our internal IHC control. Immunohistochemistry studies of 85 prostate cancer cases revealed also complete absence of SOX-4 nuclear expression in 19 (22.4%) of specimens, weak SOX-4 nuclear expression in 61 (71.7%) and high nuclear expression of SOX-4 in 5 patients (5.9%). In contrast, the glandular cells of adjacent healthy prostate areas showed lack or weak SOX-4 nuclear immunostaining and they were accepted as a negative internal control (data of internal controls not shown; instead of this, all proteins data was checked with interactive database of protein expression in healthy tissue available from https://www.proteinatlas.org [56]).

**Fig 1:**
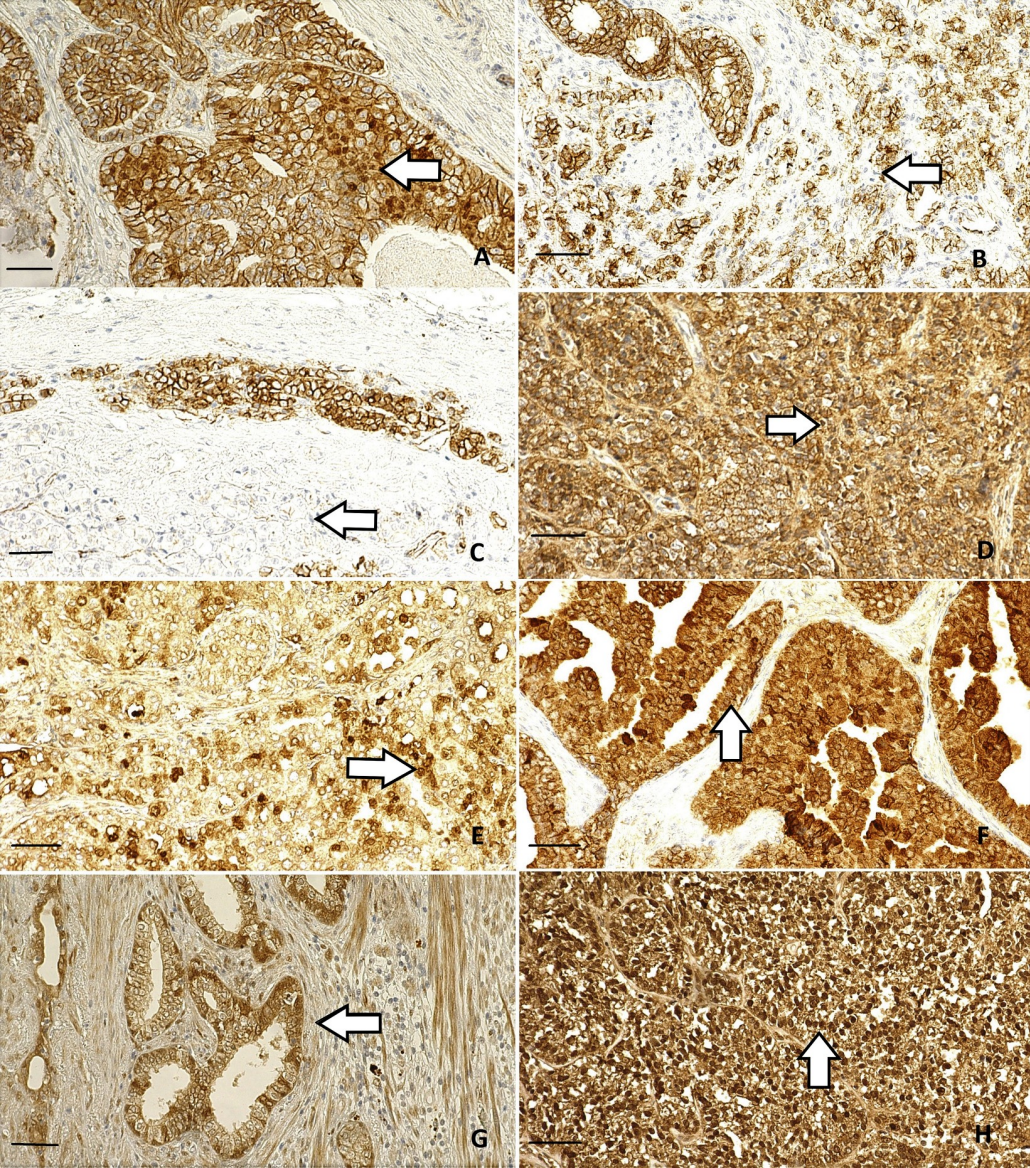
Microphotograph presenting the EMT-related proteins expression in prostate cancer tissue. Representative immunohistochemistry images presenting EMT-related proteins in TMA sections of prostate cancer tissue. A-white arrow demonstrates membrane and nuclear β-catenin expression; B-white arrow demonstrates partially reduced membrane β-catenin expression; C-white arrow demonstrates completely reduced membrane β-catenin expression; D-white arrow demonstrates completely reduced membrane E-cadherin expression; E-white arrow demonstrates the nuclear E-cadherin expression; F-white arrow demonstrates the cytoplasmic E-cadherin expression; G-white arrow demonstrates the nuclear SOX-4 expression; H-white arrow demonstrates the nuclear MIF expression. Original magnification was x10 for A-H figures. Nucleus counterstained with hematoxylin. Scale bar: 100 μm.

The examples of all types of expression pattern of β-catenin and other EMT-related proteins are presented in Fig 1. Microscopic samples analysis revealed the following: 1) The expression pattern of β-catenin in tumor cells had mostly cytoplasmic character, what was associated with a higher stage of PCa. 2) The reduced membrane β-catenin staining was related with higher Gleason pattern and it mostly occurred with Gleason pattern 4+5 and 5+5. 4) The highest membrane β-catenin expression occurred with Gleason pattern 3+3, primarily. 5) In extracapsular extension of PCa we noticed two mostly exhibited patterns, higher β-catenin expression in relation with Gleason pattern 3+3 and lower β-catenin expression in relation with Gleason pattern 4+4. 6) The highest membrane expression of E-cadherin in tumor glandular cells was closely related to Gleason pattern 3+3 and to a lesser extent with Gleason pattern 4+5 and 5+4. 7) The decreased E-cadherin expression level in samples with the highest Gleason score. 8) The nuclear-located E-cadherin expression was at the constant level.

**Fig 2:**
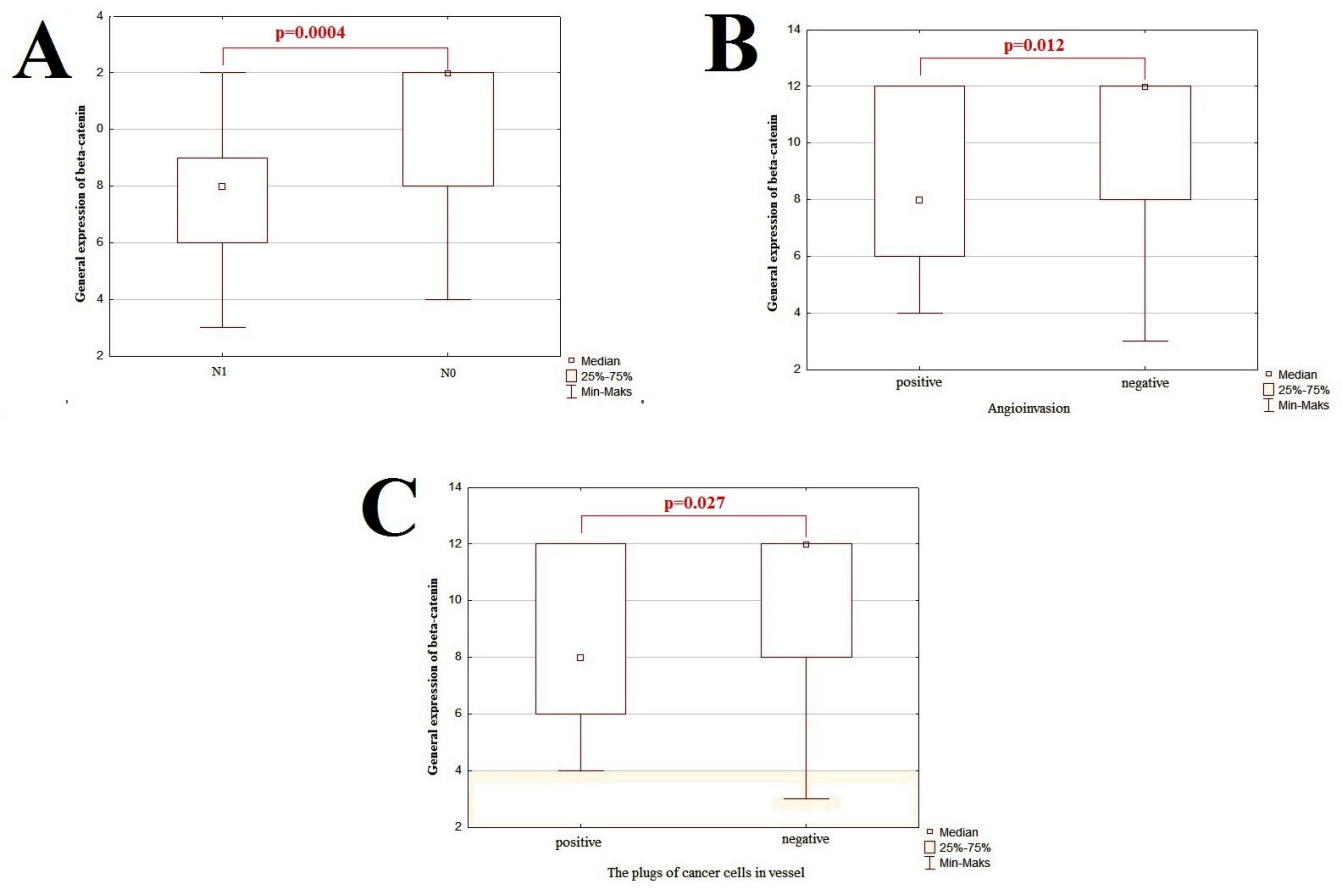
The graphic representation of results for general expression of β-catenin examined by IHC technique. The x-axis shows individual clinicopathological features and the y-axis shows IRS score values for IHC staining. The p-value <0.05 was considered as a statistically significant. Correlation between general β-catenin immunoexpression of PCa and presence of lymph node metastasis (A); angioinvasion (B) and presence of plugs of tumor cells in the vessel (C).

**Fig 3:**
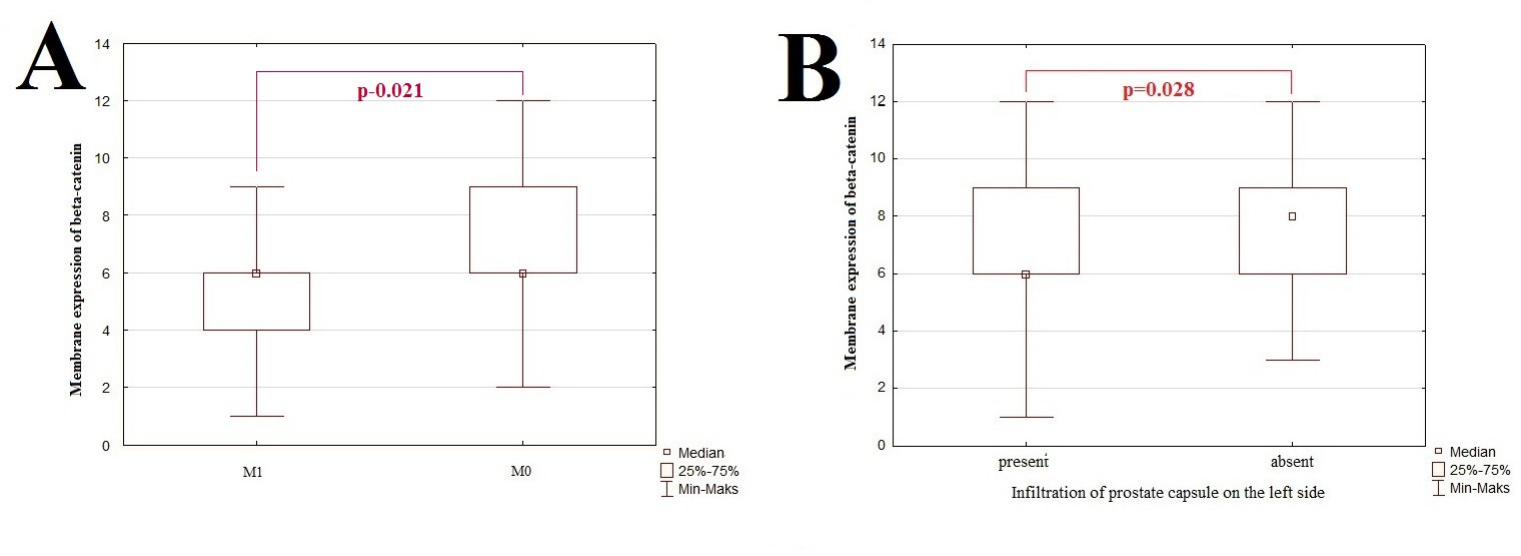
The graphic representation of results for membrane expression of β-catenin examined by IHC technique. The x-axis shows individual clinicopathological features and the y-axis shows IRS score values for IHC staining. The p-value <0.05 was considered as a statistically significant. Correlation between membrane expression of β-catenin and distant metastasis (A) and infiltration of prostate capsule on the left side (B).

**Fig 4:**
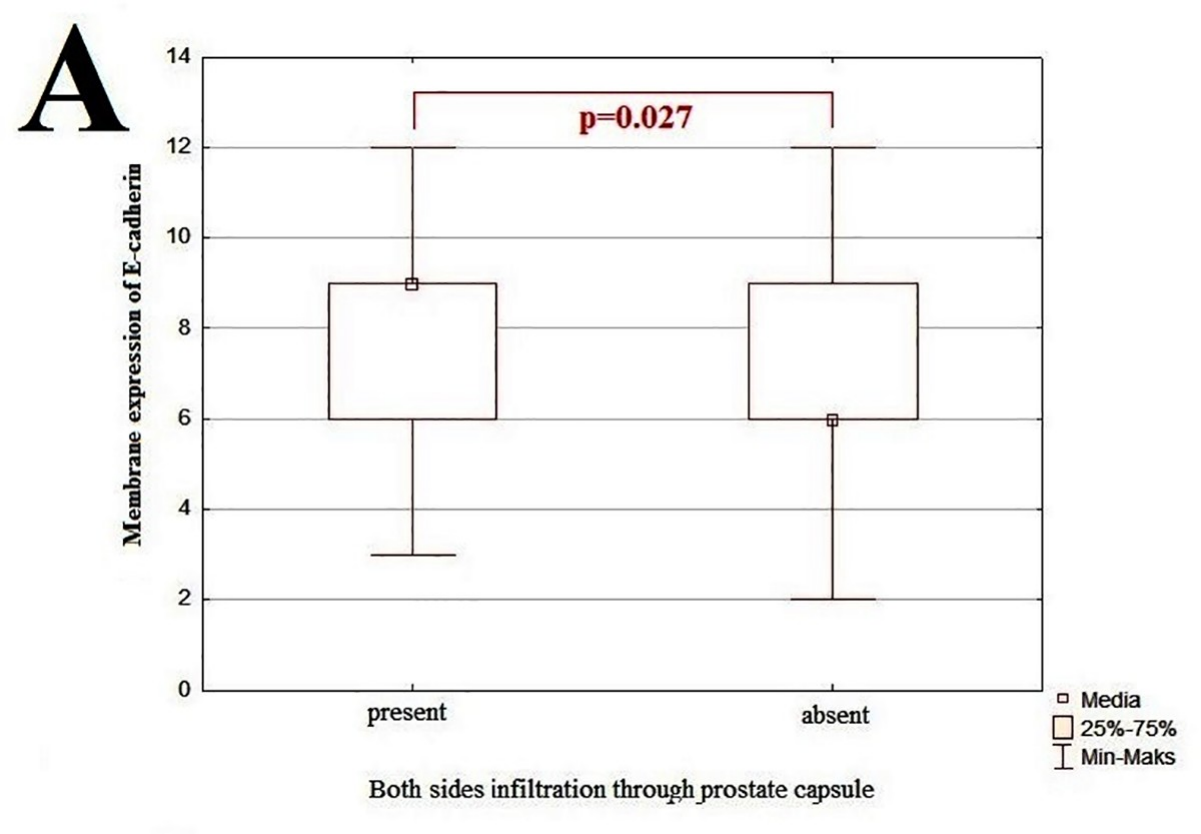
The graphic representation of results for membrane expression of E-cadherin examined by IHC technique. The x-axis shows individual clinicopathological features and the y-axis shows IRS score values for IHC staining. The p-value <0.05 was considered as a statistically significant. Correlation between membrane expression of E-cadherin and both sides infiltration of the prostate capsule (A).

**Fig 5:**
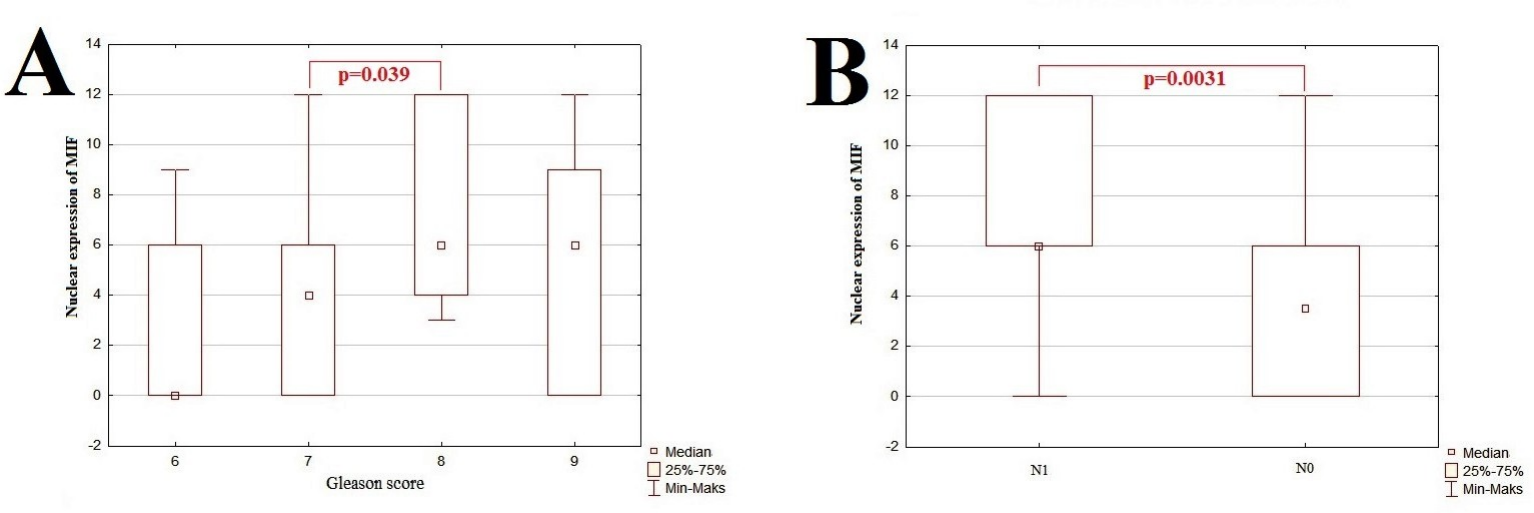
The graphic representation of results for nuclear expression of MIF examined by IHC technique. The x-axis shows individual clinicopathological features and the y-axis shows IRS score values for IHC staining. The p-value <0.05 was considered as a statistically significant. Correlation between nuclear expression of MIF and Gleason score (A) and presence of the lymph node metastasis (B).

The first aim of the current study was to determine the connection between investigated EMT-related proteins and clinicopathological features of PCa. The clinicopathological associations of EMT-related proteins were presented in Figs 2–5.

### Immunohistochemical analysis of overall β-catenin expression in PCa: Clinicopathological associations

The U Mann-Whitney analysis revealed statistically significant correlation of overall β-catenin expression with the presence of lymphovascular metastasis and angioinvasion, affirming its participation in metastasis formation. The overall expression of β-catenin level was higher in samples without infiltration of lymph nodes compared with samples with it (p<0.001; Fig 2A). We marked an involvement of overall β-catenin expression in vascular invasion. PCa cells showed the higher overall β-catenin expression in tumors with the presence of angioinvasion in regard to the tumors without it(p = 0.012; Fig 2B). Similarly, we noticed elevated overall β-catenin expression in tumor samples with plugs of cancer cells in blood vessels compared to samples without it(p = 0.027; Fig 2C).

### Immunohistochemical analysis of membrane, cytoplasmic and nuclear β-catenin expression in PCa: Clinicopathological associations

Our study revealed the participation of membranous β-catenin expression of tumor cells in formation of distant metastasis. Statistical analysis showed significant correlation of membranous β-catenin expression with the presence of distant metastasis. The membranous β-catenin expression was higher in samples without metastasis(p = 0.021; Fig 1E).

The cytoplasmic expression of β-catenin showed dependence neither with Gleason pattern and Gleason score nor with the presence of lymph node and distant metastasis and angioinvasion. The nuclear β-catenin expression remained at the constant level without any correlation with clinicopathological features. We observed that the nuclear β-catenin expression appeared in tumor areas related to Gleason pattern 4+4, primarily. Moreover, we found that tumor infiltration of left prostate gland area shows weak negative correlation with the membrane-located β-catenin (r = -0.36, p<0.05), the cytoplasm-located β-catenin (r = -0.30, p<0.05) as well as overall β-catenin expression (r = -0.33, p<0.05).

Data concerning correlations between β-catenin as well as other EMT-related proteins and main clinicopathological features was presented in two parts: Tables 3 and 4. Additionally, the data of correlations between investigated proteins and histological grading of tumor tissue which has grown through the prostate contain Tables 5 and 6.

**Table 3:** Correlation between expression of β-catenin and SOX4 and clinicopathological features of prostate cancer.

C.F.		β-catenin												SOX4		
		gen			cyt			mem			nuc			nuc		
		Q_1_	M	Q_3_	Q_1_	M	Q_3_	Q_1_	M	Q_3_	Q_1_	M	Q_3_	Q_1_	M	Q_3_
G.p.	3+3	8	12	12	4	6	9	6	9	12	2	4	4	0	0	2
	3+4	12	12	12	4	6	9	6	6	9	3	4	4	2	3	4
	3+5	6	8	12	3	3	6	3	6	6	1	4	4	2	2	2
	4+3	8	8	12	3	3	6	6	6	6	2	4	4	2	2	4
	4+4	4	7	10.5	4	6	6	5	6	6	3	4	4	1	2	3
	4+5	8	12	12	4	6	6	4	6	8	1	4	4	2	2	4
	5+4	6	8	12	4	4	12	6	6	9	2	5	6	2	2	2
	p*	ns			ns			ns			ns			ns		
G.s.	6	8	12	12	4	6	9	6	9	12	2	4	4	0	0	2
	7	8	12	12	4	6	8	6	6	9	2	4	4	2	3	4
	8	6	8	12	3	6	6	4	6	6	2	4	4	2	2	3
	9	8	12	12	4	6	6	4	6	8	2	4	4	2	2	3
	p*	ns			ns			ns			ns			ns		
T	3	12	12	12	6	6	6	8	8	8	4	4	4	2	3	4
	3a	8	12	12	4	6	8	6	6	9	2	4	4	0	2	3
	3b	8	12	12	3	6	6	6	6	8	2	4	4	2	3	4
	4	7	10	12	4.5	6	9	6	7.5	9	1.5	3	5	2	2.5	3.5
	p*	ns			ns			ns			ns			ns		
N	0	8	12	12	4	6	6	6	6	9	2	4	4	2	2	4
	1	6	8	9	3	4	6	4	6	6	2	4	4	2	2	3
	p**	0.0004			ns			ns			ns			ns		
M	0	8	12	12	4	6	6	6	6	9	2	4	4	2	2	4
	1	8	9	12	4	6	6	4	6	6	1	4	4	0	2	3
	p**	ns			ns			0.021			ns			ns		
Ang	(-)	8	12	12	4	6	6	6	6	9	2	4	4	2	2	4
	(+)	6	8	12	3	4	6	6	6	9	2	4	4	2	2	2
	p**	0.012			ns			ns			ns			ns		
Plu	(-)	6	12	12	3	6	6	6	6	9	2	4	4	2	2	4
	(+)	6	8	12	3	5	8	6	6	9	2	4	4	2	2	3
	P**	0.027			ns			ns			ns			ns		

SOX4- Transcription factor SOX-4; gen- general expression; cyt- cytoplasmic expression; mem- membrane expression; nuc- nuclear expression; C.F.- Clinical Feature; G.p.- Gleason pattern; G.s.- Gleason score; T- T stage according to TNM classification; N- N stage according to TNM classification; M- M stage according to TNM classification; Ang- Presence of the angioinvasion; Plu- Presence of plugs of cancer cells in vessel, p-v- p-value; (+) -present; (-)–absent. Q1—the first quartile; M- Median; Q3—the third quartile; ns- non significant

* Kruskal-Wallis Test

** Mann–Whitney U test.

**Table 4:** Correlation between expression of MIF and E-cadherin and clinicopathological features of prostate cancer.

C.F.		MIF						E-cadherin											
		cyt			nuc			gen			mem			Cyt			nuc		
		Q_1_	M	Q_3_	Q_1_	M	Q_3_	Q_1_	M	Q_3_	Q_1_	M	Q_3_	Q_1_	M	Q_3_	Q_1_	M	Q_3_
G.p.	3+3	8	8	8	0	0	6	12	12	12	6	9	12	8	8	12	4	4	6
	3+4	8	12	12	0	4	6	12	12	12	6	9	9	8	12	12	6	6	6
	3+5	12	12	12	9	9	12	12	12	12	6	9	9	12	12	12	6	8	9
	4+3	8	12	12	0	4	6	8	12	12	6	9	9	8	12	12	4	6	6
	4+4	6	10	12	3	5	9	8	8	12	5	6	9	8.5	10.5	12	6	6	7.5
	4+5	8	12	12	0	6	9	12	12	12	6	6	9	8	8	9	4	6	8
	5+4	3	12	12	0	6	12	12	12	12	6	9	9	12	12	12	6	6	9
	p*	ns			ns			ns			ns			ns			ns		
G.s.	6	8	8	8	0	6	6	12	12	12	6	9	9	8	12	12	4	6	6
	7	8	12	12	0	4	6	12	12	12	6	9	9	8	12	12	4	6	6
	8	8	12	12	4	6	12	8	12	12	6	6	9	9	12	12	6	6	9
	9	8	12	12	0	6	9	12	12	12	6	6	9	8	9	12	6	6	8
	p*	ns			0.039			ns			ns			ns			ns		
T	3	8	8	8	0	0	0	12	12	12	9	10.5	12	6	7.5	9	6	6	6
	3a	8	10	12	0	4	6	12	12	12	6	8	9	8	12	12	4	6	8
	3b	8	12	12	0	6	9	9	12	12	6	9	9	8	12	12	4	6	6
	4	12	12	12	0	3	9	12	12	12	6	7.5	9	9	10.5	12	5	6	7
	p*	ns			ns			ns			ns			ns			ns		
N	0	8	12	12	0	3.5	6	12	12	12	6	9	9	8	12	12	4	6	6
	1	8	12	12	6	6	12	8	12	12	6	6	9	8	9	12	4	6	6
	p**	ns			0.003			ns			ns			ns			ns		
M	0	8	12	12	0	4	6	12	12	12	6	9	9	8	12	12	6	6	6
	1	6	12	12	3	6	9	8	12	12	6	6	9	8	9	9	4	4	6
	p**	ns			ns			ns			ns			ns			ns		
Ang	(-)	8	12	12	0	4	6	12	12	12	6	9	9	8	12	12	4	6	6
	(+)	6	8	12	0	6	9	8	12	12	6	9	9	8	12	12	6	6	6
	p**	ns			ns			ns			ns			ns			ns		
Plu	(-)	6	12	12	0	4	6	8	12	12	6	9	9	8	12	12	6	6	6
	(+)	6	10	12	0	6	9	8	12	12	6	9	9	8	12	12	6	6	6
	P**	ns			ns			ns			ns			ns			ns		

MIF- Macrophage migration inhibitory factor; gen- general expression; cyt- cytoplasmic expression; mem- membrane expression; nuc- nuclear expression; C.F.- Clinical Feature; G.p.- Gleason pattern; G.s.- Gleason score; T- T stage according to TNM classification; N- N stage according to TNM classification; M- M stage according to TNM classification; Ang- Presence of the angioinvasion; Plu- Presence of plugs of cancer cells in vessel, p-v- p-value; (+) -present; (-)–absent. Q1—the first quartile; M- Median; Q3—the third quartile; ns- non significant

* Kruskal-Wallis Test

** Mann–Whitney U test.

**Table 5:** Correlations between β-catenin, SOX-4 and invasion area and histological grading of tumor tissue which has grown through the prostate.

C.F.			β-catenin												SOX4		
			gen			cyt			mem			nuc			nuc		
			Q_1_	M	Q_3_	Q_1_	M	Q_3_	Q_1_	M	Q_3_	Q_1_	M	Q_3_	Q_1_	M	Q_3_
oPC	R	(+)	8	12	12	4	6	6	6	6	9	2	4	4	2	2	3
		(-)	8	12	12	3	6	8	6	6	9	2	4	4	0	2	3
		p*	ns			ns			ns			ns			ns		
	L	(+)	8	12	12	3	6	6	6	6	9	2	4	4	2	2	3
		(-)	8	12	12	4	6	7	6	8	9	2	4	4	0	2	4
		p*	ns			ns			0.028			ns			ns		
	B	(+)	8	12	12	4	6	6	4	6	9	4	4	4	2	2	3
		(-)	8	12	12	3	6	8	6	6	9	2	4	4	0	2	3
		p*	ns			ns			ns			ns			ns		
PA	R	(+)	8	12	12	4	6	6	6	6	9	3	4	4	2	2	4
		(-)	8	8	12	3	4	6	6	6	6	2	4	4	0	2	3
		p*	ns			ns			ns			ns			ns		
	L	(+)	12	8	12	6	3	6	6	6	9	4	2	4	2	2	3
		(-)	12	12	12	6	4	8	8	6	9	4	4	4	2	2	4
		p*	ns			ns			ns			ss			ns		
	B	(+)	8	12	12	3	6	6	6	6	9	2	4	4	2	2	3
		(-)	8	12	12	4	6	6	6	6	9	2	4	4	0	2	4
		p*	ns			ns			ns			ns			ns		
SV	R	(+)	8	12	12	3	6	6	6	6	8	2	4	4	2	2.5	4
		(-)	8	12	12	4	6	6	6	6	9	2	4	4	0	2	3
		p*	ns			ns			ns		8	ns			ns		
	L	(+)	8	12	12	3	6	6	6	6	9	2	4	4	2	3	4
		(-)	8	12	12	4	6	8	6	6		2	4	4	0	2	3
		p*	ns			ns			ns			ns			ns		
	B	(+)	12	8	12	6	3	6	6	6	8	4	2	4	3	2	4
		(-)	12	8	12	6	4	6	6	6	9	4	3	4	2	0	3
		p*	ns			ns			ns			ns			ns		
G.p.*	3+3		8	12	12	4	6	8.5	6	8	10.5	2	4	4	2	2	4
	3+4		12	12	12	6	9	9	6	6	9	4	4	6	3	4	4
	4+3		10	12	12	4	5	7.5	6	6	7.5	3	4	4	0	1.5	5.5
	4+4		8	10.5	12	3	6	6	6	6	6	2	4	4	2	2	3
	4+5		8	12	12	3	6	6	2	4	8	0	4	4	0	2	3
	5+4		12	12	12	4	4	4	6	6	6	4	4	4	2	2	2
	5+5		8	8	10	3.5	4	6.5	4	6	6	1	3	4	1	2	3
	p*		ns			ns			ns			ns			ns		
G.s.*	6		8	12	12	4	6	8.5	6	8	10.5	2	4	4	2	2	4
	7		12	12	12	6	6	9	6	6	9	4	4	4	0	3	4
	8		8	10.5	12	3	6	6	6	6	6	2	4	4	2	2	3
	9		8	12	12	3.5	5	6	3	5	7	2	4	4	1	2	2.5
	10		8	8	10	3.5	4	6.5	4	6	6	1	3	4	1	2	3
	p*		ns			ns			ns			ns			ns		

SOX4- Transcription factor SOX-4; gen- general expression; cyt- cytoplasmic expression; mem- membrane expression; nuc- nuclear expression; Q1—the first quartile; M- Median; Q3—the third quartile, iA- invaded Area; oPC- outside the prostate capsule, PA- the apex of prostatic gland; SV- seminal vesicles; R- on the right side, L- on the left side; B- on the both sides p-v- p-value; (+) -invasion; (-)–without invasion.; ns- non significant; G.p.- Gleason pattern; G.s.- Gleason score; p-v- p-value; Q1—the first quartile; M- Median; Q3—the third quartile; ns- non significant

* Kruskal-Wallis Test.

**Table 6:** Correlations between MIF and E-cadherin and invasion area and histological grading of tumor tissue which has grown through the prostate.

Features			MIF						E-cadherin											
			cyt			nuc			Gen			Mem			cyt			nuc		
			Q_1_	M	Q_3_	Q_1_	M	Q_3_	Q_1_	M	Q_3_	Q_1_	M	Q_3_	Q_1_	M	Q_3_	Q_1_	M	Q_3_
oPC	R	(+)	8	12	12	0	4	6	12	12	12	6	8.5	9	8	9	12	4	6	6
		(-)	8	8	12	0	3	9	12	12	12	6	9	9	8	12	12	4	6	6
		p*	ns			ns			ns			ns			ns			ns		
	L	(+)	8	12	12	0	4	9	12	12	12	6	9	9	8	12	12	4	6	6
		(-)	8	8	12	0	1.5	5	12	12	12	6	9	9	8	12	12	6	6	6
		p*	ns			ns			ns			ns			ns			ns		
	B	(+)	12	12	12	3	5	9	8	12	12	6	6	9	8	9	12	4	6	6
		(-)	8	8	12	0	3	6	12	12	12	6	9	9	8	12	12	6	6	6
		p*	0.022			ns			ns			0.027			ns			ns		
PA	R	(+)	8	12	12	0	4	6	12	12	12	6	9	9	8	12	12	4	6	6
		(-)	4	8	12	0	4	9	12	12	12	6	9	9	8	12	12	4	6	8
		p*	ns			ns			ns			ns			ns			ns		
	L	(+)	12	8	12	4	0	6	12	8	12	9	6	9	12	8	12	6	4	6
		(-)	8	8	12	3.5	0	9	12	12	12	9	6	12	12	8	12	6	6	6
		p*	ns			ns			ns			ns			ns			ns		
	B	(+)	8	12	12	0	4	6	8	12	12	6	9	9	8	12	12	4	6	6
		(-)	8	8	12	0	4	9	12	12	12	6	9	9	8	12	12	4	6	6
		p*	ns			ns			ns			ns			ns			ns		
SV	R	(+)	8	12	12	0	5	9	8	12	12	6	9	9	8	10,5	12	4	6	6
		(-)	8	12	12	0	4	6	12	12	12	6	8	9	8	12	12	4	6	6
		p*	ns			ns			ns			ns			ns			ns		
	L	(+)	8	12	12	0	4	6	9	12	12	6	9	9	9	9	12	6	8	9
		(-)	8	12	12	0	4	6	12	12	12	6	9	9	8	12	12	6	6	9
		p*	ns			ns			ns			ns			ns			ns		
	B	(+)	12	8	12	4	0	9	12	8	12	9	6	9	9	8	12	4	6	6
		(-)	12	8	12	4	0	6	12	12	12	9	6	9	12	8	12	4	6	6
		p*	ns			ns			ns			ns			ns			ns		
G.p.*	3+3		8	10	12	0	3.5	5	12	12	12	6	9	9	8	12	12	5	6	6
	3+4		8	12	12	0	0	4	12	12	12	8	9	12	8	12	12	4	6	6
	4+3		3	4	6	0	4.5	9	8	10	12	6	7.5	9	8	10	12	5	6	6
	4+4		8	12	12	3	5	9	8	12	12	6	9	9	8	12	12	4	6	6
	4+5		8	12	12	0	6	9	12	12	12	4	6	9	8	8	9	4	6	9
	5+4		12	12	12	6	6	6	12	12	12	6	6	6	12	12	12	9	9	9
	5+5		3.5	6	8	0	0	0	8	10	12	4.5	7	8.5	9	12	12	5	9	12
	p*		ns			ns			ns			ns			ns			ns		
G.s.*	6		8	10	12	0	3.5	5	12	12	12	6	9	9	8	12	12	5	6	6
	7		4	8	12	0	0	9	12	12	12	6	9	9	8	12	12	4	6	6
	8		8	12	12	3	5	9	8	12	12	6	9	9	8	12	12	4	6	6
	9		8	12	12	0	6	9	12	12	12	5	6	9	8	8	10.5	5	6	9
	10		3.5	6	8	0	0	0	8	10	12	4.5	7	8.5	9	12	12	5	9	12
	p*		ns			ns			ns			ns			ns			ns		

MIF- Macrophage migration inhibitory factor; gen- general expression; cyt- cytoplasmic expression; mem- membrane expression; nuc- nuclear expression; Q1—the first quartile; M- Median; Q3—the third quartile, iA- invaded Area; oPC- outside the prostate capsule, PA- the apex of prostatic gland; SV- seminal vesicles; R- on the right side, L- on the left side; B- on the both sides p-v- p-value; (+) -invasion; (-)–without invasion.; ns- non significant; G.p.- Gleason pattern; G.s.- Gleason score; p-v- p-value; Q1—the first quartile; M- Median; Q3—the third quartile; ns- non significant

* Kruskal-Wallis Test.

### Immunohistochemical analysis of membrane and cytoplasm E-cadherin expression in PCa: Clinicopathological associations

The analysis of protein expression and clinicopathological features showed that the cancer invasion of the right surface of prostate gland negatively corresponded with cytoplasm-located E-cadherin expression (r = -0.25, p<0.05) at low level, whereas tumor invasion of the left surface of the prostate gland was negatively associated with the membrane-located expression of E-cadherin (r = -0.26, p<0.05) at low level. Membrane expression of E-cadherin negatively corresponded with prostate weight (r = -0.23, p<0.05) at low level. In the specimens where tumor infiltrated the both, right and left side of the gland, we observed significant decrease of membrane E-cadherin expression level, than in specimens where tumor infiltrate only one side of gland (p = 0.019; Fig 1F). Furthermore, the cancer cells invasion of the prostate gland left area corresponded with the overall β-catenin expression at poor negative level (r = -0.33, p<0.05).

### Immunohistochemical analysis of MIF and SOX-4 expression in PCa: Clinicopathological associations

We found a statistically significant increase in nuclear expression of MIF between Gleason score 7 and 8 (p = 0.039; Fig 1G). Moreover, we noted higher expression of nuclear MIF in samples with the presence of lymphovascular metastasis-N1 in comparison to samples without lymphovascular metastasis-N0 (p = 0.003; Fig 1H). We displayed that age has a negative association with nuclear expression of MIF (r = -0.24, p<0.05) at weak level. Also, in PCa with infiltration both right and left area of the gland, we revealed a significant increase in the cytoplasm-located MIF (p = 0.011) and nuclear-located MIF (p = 0.044), than in samples where tumor infiltrate only one side of the gland.

Additionally, we showed that SOX-4 has no correlation with any clinicopathological features and it is our unexpected result.

### The co-expressions between β-catenin, E-cadherin, SOX-4 and MIF proteins

**Fig 6:**
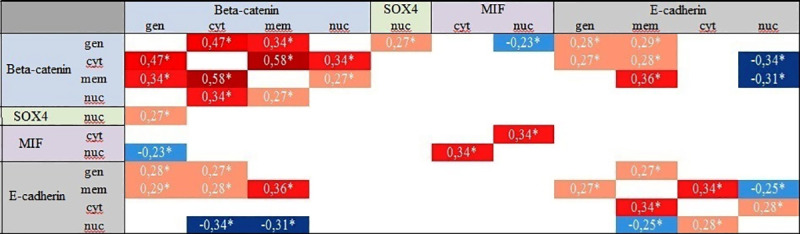
Correlation heat map of proteins expression data measured by immunohistochemistry. Spearman’s rank correlation coefficient was calculated among 110 compilations of protein expression levels. The degrees of correlation are color-coded. The color key designates the correlation was statistically significant. As a statistically significant was considered p-value < 0.05. Red color shades indicate a positive proteins correlation, whereas blue color shades indicate negative proteins correlation. β-catenin, Transcription factor SOX-4, Macrophage migration inhibitory factor and E-cadherin were designated Beta-catenin, SOX-4, MIF, E-cadherin, respectively. Protein correlations are presented taking into account cellular localization. General, cytoplasmic, membrane and nuclear expressions were signed as gen, cyt, mem, nun, respectively.

The interactions between investigated proteins provide valuable information about the natures of the interacting proteins. Our protein co-expression analysis suggests novel insight into β-catenin-E-cadherin-SOX-4-MIF inter-factor dependence. Correlations between individual proteins were measured based on increasing or decreasing levels in various cell locations. The correlations between studied proteins were included in Fig 6.

Regarding to the Spearman rank coefficient, the positive association was detected between the both overall β-catenin and E-cadherin expression (r = 0.28, p<0.05) and also between the both membrane expression level of β-catenin and E-cadherin (r = 0.36, p<0.05).

Next, we focused on checking the relation of SOX-4 transcription factor as well as MIF cytokine with other EMT-related proteins to show their possible impact on metastasis initiation. Consequently, we displayed a weak positive correlation between total β-catenin protein expression with level of nuclear SOX-4 protein (r = 0.27; p<0.05) and weak negative correlation of total β-catenin expression with level of nuclear MIF protein expression (r = -0.23), p<0.05). Accordingly, we supposed that MIF and SOX-4 factors may cooperate in acquiring invasive properties by tumor cells in prostate malignancies. However, we were unable to confirm immunohistochemically this relationship.

## Discussion

Loss of cell-cell adhesion capacity permits the malignant cells to detach and leave the primary tumor location. In turn, changes in cell-matrix interaction allow the tumor cells to occupy the neighbouring stroma. Saha et al., have detected homogenous membrane expression of E-cadherin and β-catenin in benign prostatic hyperplasia (BPH) at similar frequency pattern, whereas in primary PCa, it was significantly reduced. Interestingly, a similar expression and frequency pattern of E-cadherin and β-catenin as in BPH was presented in invasive cells of PCa with bone metastasis [57]. Likewise, in our study we observed a particularly reduced E-cadherin and β-catenin membrane pattern in relation to Gleason pattern 4+5 or 5+4 and 4+5 or 5+5, respectively. Invasive cells that exceeded the capsule of the prostate gland exhibited higher membrane β-catenin pattern in areas with Gleason pattern 3+3 and lower expression in areas with Gleason pattern 4+4. The invasive potential of malignant cells is required to dissociate from the primary tumor cluster. Next, displaced tumor cells may invade surrounding or distant stroma and no longer require invasive phenotype. The enhanced β-catenin nuclear translocation may activate or deactivate specific targets and stimulate a transcription factors changing cell phenotype from epithelial into mesenchymal [9, 12]. In current study, the nuclear β-catenin expression remained at the constant level without any correlation with clinicopathological features. Interestingly, we observed that translocation of β-catenin to the nucleus was present primarily in tumor areas related to Gleason pattern 4+4. The increasing aggressiveness of tumors seems to be connected with the redistribution of beta-catenin in tumor cells. This event was related to the passage of membrane-located β-catenin to the intracellular area of the tumor cells. We revealed that in our tumor samples there were only hotspots showing the transition of β-catenin from cell to cell boundaries to the cytoplasm. Probably, during the assessment of protein expression in individual sample we should focus on evaluating the several specific hotspots to standardize the results.

In most epithelial cancers the both E-cadherin and β-catenin repression is followed by transcriptional silencing provided by cooperating with EMT transcription factors (EMT-TFs). The SOX-4 may be one of the numerous EMT-TFs necessary for initiation of cytoskeletal rearrangements and supporting the multi-step metastasis process. Here, we report a weak correlation between the total expression of β-catenin and nuclear expression of SOX-4 protein (r = 0.27; p<0.05). Indeed, it appeared in evidence that β-catenin level was weak negative correlated with the nuclear MIF (r = -0.23, p<0.05). It demonstrated that MIF as a proinflammatory cytokine may actively participate in stimulating signal transduction pathways involved in EMT, controlling the acquisition of invasiveness features by tumor cells and facilitating metastasis processes. Previous studies have provided that E-cadherin, as same as β-catenin, effectively influences on enhancing tumor progression and has effect on converting to its metastatic form [58–60]. Chang et al. showed that metastasis of prostate cancer cells to lymph nodes expressed less E-cadherin level than primary PCa [61]. Multiple studies have linked loss of membrane E-cadherin in PCa associated with the acquisition of invasive properties [62]. Jaggi et al., in their semi-quantitative immunohistochemistry analysis, have shown the reduction of immunostaining for the both β-catenin and E-cadherin in PCa compared to normal glandular epithelium. The decrease of β-catenin and E-cadherin expressions were accompanied by rising tumor invasiveness [63, 64]. Moreover, Horvath et al. demonstrated that low level of nuclear β-catenin expression of the tumor cells in the early diagnosed PCa is a marker related with highly poor prognosis in patients, among who the prognosis seemed to be promising and those patients were classified into radical prostatectomy [65]. Admittedly, we did not find any correlations between E-cadherin and the presence of lymph nodes or distant metastasis, however it is indisputable that loss of E-cadherin is highly associated with metastasis capability in PCa. Otherwise, we revealed that reducing of the membranous E-cadherin in prostate epithelium may influence the enlargement of gland neoplasm. Some studies have demonstrated a direct interaction between SOX-4 and β-catenin [25, 66]. The SOX-4 transcription factor has been shown to enhance the cell proliferation in SW480 colon cancer cell lines through stabilization of β-catenin activated aberrant Wnt signaling pathways [25]. Our study suggested the expression level of β-catenin was probably activated by a transcription factor SOX-4, leading to changes in subcellular localization of β-catenin in PCa. Stimulation of β-catenin located in cell membrane via SOX-4 transcription factor may lead to translocation of β-catenin to cytoplasm or partially to the nucleus, initiating the cell phenotype transformation from epithelial to mesenchymal state. Hence, knockdown of endogenous SOX-4 notably decreases the migration and invasion ability of tumor cells in vitro, thereby leads to reverse of the EMT cascade by increasing E-cadherin [67]. Birdal et al. have demonstrated in the in vivo study that Sox4 deletion affects on decreasing of active β-catenin level [68]. It is consistent with our findings confirming that SOX-4 may be responsible for regulation of the β-catenin signaling pathway, by influencing on β-catenin in tumor cells. Makoto and colleagues have shown that SOX-4 has a positive impact on the β-catenin signal transduction through changes in TCF4 expression during the morular differentiation of endometrial carcinoma cells, thus providing the proliferation arrest [69]. There are a lot of publications showing the role of SOX-4 in promotion of EMT and confirming its strong involvement in acquisition of aggressiveness and invasiveness. Moreover, the SOX-4 transcription factor may be involved in controlling many issues of tumor expansion in different types of cancer. Many scholars have confirmed the participation of SOX-4 transcriptional factor in the PCa progression showing its relationship with high Gleason score (p = 0.009) and the presence of distant metastasis (p = 0.023) [70]. The study on colon cancer, where the nuclear SOX-4 overexpression was closely corresponded with tumor invasion and distant metastasis [71], has been proved the crucial role of SOX-4 protein in tumor development and EMT initiation. Consequently, Liu P. et al. in their study on PCa cells also have shown that the both at the mRNA and protein level, SOX-4 was highly correlated with Gleason score [72]. Unexpectedly, our Spearman correlation analysis revealed no association between the SOX-4 protein and any clinicopathological features. We considered the discrepancies between results of current and previous studies as a consequence of dissimilarity of applied protein detection techniques, different antibody clones available to protein detection, IHC visualization kits and multifocal character of PCa. The high genomic diversity degree and morphological heterogeneity of prostate neoplasm make it difficult to obtain a repeatable results of immunohistochemical staining and microscopic analysis. A thin tissue cores may not be representative for whole cancer areas.

Our next aim was to investigate the role of inflammation in promotion of EMT in prostate adenocarcinoma. Recent studies focused on inflammation-induced epithelial cell injury have shown the fundamental role of inflammation in initiation and progression of prostate cancer [73]. MIF is one cytokine with T lymphocyte origin that presumably participates in an immune mechanism related to cancer microenvironment [74]. Activated T cells secrete a MIF factor that has impact on cell mitosis and initiate transformation of cells to malignant phenotype, thereby facilitating tumor progression [74, 75]. Meyer-Siegler and Hudson in their study have indicated that MIF excreted by metastatic cells may manage functioning of macrophages and secretion of cytokines [76]. Also, MIF molecules may participate in accumulation of macrophages related to PCa and affect on tumor maintenance [76]. MIF manifests its pro-inflammatory nature, showing high expression levels in tissues with chronic inflammation areas, such as hepatitis, gastritis and pancreatic [77]. It is worth to notice that many previous studies have shown increased MIF expression levels associated with benign prostate hyperplasia, induced by chronic conditions [78]. Nevertheless MIF has been found as a cytokine, strongly corresponded with prostate adenocarcinoma and disease progression.

MIF can activate EMT cascade through E-cadherin downregulation and N-cadherin overexpression, leading to form secondary site tumors [79]. Our study revealed that translocation of the MIF factor to the nucleus could enable β-catenin upregulation and activate the EMT process. Probably, stimulated and altered β-catenin expression, both total and membrane, inversely interacts with E-cadherin accumulated in the nucleus and consequently leads to a reduction in tumor cell-to-cell adhesion.

Funamizu et al. have used mice cells to show significant overexpressed MIF in progressive tumor growth compared to the control cells and thus highlighted the role of MIF in accelerating progression and metastasis of pancreatic ductal adenocarcinomas [39]. Our study exhibited that nuclear MIF expression is a strong predictor of lymph node metastasis, therefore MIF may act as a mediator, modulated to accelerate the tumor progression to its aggressive state. To the best of our knowledge, only present study focused the attention on examination of the both cytoplasmic and nuclear expression of MIF in PCa. Contrary to our results, suggesting that PCa patients with high nuclear MIF expression may be assigned a poor prognosis, Kamimura et al. have discovered that patients with lung cancer, without confirmed nuclear expression of MIF factor in tumor cells, had poorer prognosis than cases with confirmed MIF nuclear expression [50]. Similarly to our findings, Ren et al. have revealed that rMIF fraction contributes the invasion and migration of HCC cells by an in vitro cell migration assay [80]. Additionally, in prostate neoplasm, MIF factor has been found precisely corresponding with tumor progression and metastasis [76]. Lately, MIF has been described as a novel therapeutic target against metastatic triple-negative breast cancer (TNBC). MIF inhibitor CPSI-1306 may silence TNBC growth and metastasis through activating apoptosis [81]. Taken as a whole, current and previous results seem to indicate that MIF molecule might be an important checkpoint inducing transformation into an aggressive and metastatic form of cancer. Interestingly, MIF is a part of several clinical trials. One of this trials was phase III interventional study with attendance of 60 PCa participants, titled “A Double-Blind, Randomised, Placebo-Controlled Study of the Effect of Transdermal Nitroglycerin (Glyceryl Trinitrate; GTN) Therapy on Biomarkers of Immune Escape in Men With Biochemical Recurrence of Prostate Cancer After Primary Therapy” (ClinicalTrials.gov Identifier: NCT01704274). For evaluating the effect of intervention with Glyceryl Trinitrate doses as a primary outcome measures was applied inter alia changes of MIF biomarker level.

Vecchio and colleagues have indicated the existence of association between level of MIF factor and cellular differentiation in untreated PCa, showing that MIF expression was stronger in low-grade adenocarcinoma (GS≤6) than in high-grade adenocarcinoma (GS≥7) [82]. In another study by Chen et al., specimens with a Gleason score of 7 was more frequently present enhanced MIF expression than the specimens with a Gleason score of 6 [83]. We showed that high expression of nuclear MIF was more likely associated in prostate tumor areas with a Gleason score of 8 than in those with a Gleason score of 7. Thus, our results support the hypothesis that nuclear MIF overexpression may predict poor prognosis for PCa patients.

Moreover, we found that upregulation of the cytoplasmic MIF, produced by the prostatic cancer epithelium, participates in the infiltrating of both the right and left zone of prostate cancer gland. Verjans et al., have shown MIF expression as significantly associated with tumor size in breast cancer (p = 0.007), where size of tumor above 2 cm corresponded with cancer progression and manifested in low MIF expression with IRS score less than 4 [43]. Admittedly, we did not confirm association between tumor size and MIF deregulation, however we highlighted its role in tumor growth. Meyer-Siegler et al. using more advanced molecular techniques, like Slot blot analysis of RNA in PCa, have demonstrated that elevated MIF expression is strongly corresponded with mesenchymal phenotype of prostate disease. They have shown that MIF secretes by PCa cells may play an important role in the multi-step process of metastatic cascade [76]. The enhanced immunostaining of MIF protein in prostate malignancies and its correlation with lymphovascular metastases suggested that MIF may contribute to the acceleration of PCa progression through involvement in the initiation of lymph node metastases. Bando et al., have found significant negative dependence between deregulation of MIF factor related to tumor microenvironment, detected by ELISA test and presence of nodal metastasis in breast cancer [45]. Our results are also consistent with Pei et al.’ study of MIF and DJ-1 protein in Nasopharyngeal Carcinoma (NPC). They have shown that high expression of MIF protein was significantly correlated with advanced clinical stage, nodal metastasis and poor prognosis, thus had influence on initiation of cell invasion and metastasis in NPC [84].

Translocation of MIF from cytoplasm to the nucleus may play a critical role in PCa progression and seems to be important for tumor cell growth and invasion. Nuclear redistribution of MIF factors may interact with deregulation of transcription factors, such as SOX-4 leading to facilitate the process of EMT. In our study, we did not find any correlations between SOX-4 and MIF proteins, that is inconsistent with our hypothesis, in which we assumed that SOX-4 activity could be controlled by upregulation of MIF during prostate tumor progression. However, the immunohistochemical analysis in this study is insufficient to confirm this relationship. Using more advanced molecular tools, we could get satisfying results. A study on correlation between MIF and SOX-4 to check the potential role of SOX-4 as a downstream target of MIF overexpression in PCa has not been performed yet. Nevertheless, we have found the study with other SOX family members and MIF factors. In the study performed by Yuan et al., using among others the luciferase reporter assay in a 293T cell line and chromatin immunoprecipitation (ChIP)-PCR assay, it has shown that overexpression of MIF is responsible for transcriptional activity of SOX-9 during the chondrogenesis and osteogenesis, [85]. Moreover, Shigeki et al. have shown that MIF molecules regulate the Sox6 expression in mouse NSPCs via the interaction with STAT3 molecules [86]. However, our study did not confirm that MIF factor may impact on the activity of SOX-4 protein. We did not find any significant association between the both nuclear or cytoplasmic MIF expression and nuclear expression of SOX-4 protein. We want to point out that the study at alterations in protein levels using immunohistochemistry method do not always correlate with the results reached by other techniques such as cell culture or molecular techniques.

## Conclusion

Many previously carried out studies as well as the current research emphasize the significant role of β-catenin, SOX-4, MIF and E-cadherin in malignant transformation of prostate tumor cells and confirmed their attendance in one of the initial metastasis steps—epithelial–mesenchymal transition (EMT). Immunohistochemical study may not provide the final evidence that SOX-4 is not a downstream target of MIF, therefore definitive conclusions cannot be drawn due to insufficient knowledge. Advanced studies using molecular biology techniques are required to confirm the direct or indirect protein-protein interactions. At this stage, we can corroborate protein inter-relationships based on expression patterns of studied factors and outline the direction for further research. However, our results seem promising and strongly highlight the potential role of MIF as a treatment option in metastatic PCa. The MIF targeting could help to reverse disease progression at both early and advanced stages in future.

## Supporting information

S1 Table The additional values of significant correlations between protein expression and clinicopathological features. (DOCX)

S2 Table Comparison of significant values for Mann-Whitney U test. (DOCX)

## Abbreviations

PCa: prostate cancer
SEER: Surveillance, Epidemiology, and End Results Program
ECM: degrading enzymes: extracellular matrix degradation enzymes
EMT: epithelial-mesenchymal transition
AJ: adherens junctions
GSK3β: glycogen synthase kinase 3β
HMG: high-mobility-group
TCF: T-cell factor
LEF: lymphoid enhancer factor
SOX-4: SRY-Box Transcription Factor 4
SRY: sex-determining region Y
DBD: DNA-binding domain
MIF: Macrophage migration inhibitory factor
TPOR: thiol protein oxidoreductase
FFPE: formalin-fixed paraffin embedded
AJCC: American Joint Committee on Cancer
GS: Gleason score
TMA: tissue microarray
IHC: immunohistochemistry
IRS: immunoreactive score
25p: 25^th^ percentile
M: median
75p: 75^th^ percentile
BPH: benign prostatic hyperplasia
EMT-TFs: EMT transcription factors
TNBC: triple-negative breast cancer
NPC: nasopharyngeal carcinoma

## References

[1] Rawla P. Epidemiology of prostate cancer. Pathol Epidemiol Cancer. 2019;10(2):107–25. doi: 10.14740/wjon1191 31068988 PMC6497009

[2] Institute NNC. Cancer Facts & Figures 2020. 2020. [Cited 2020 March 17]. Available from: https://www.cancer.org

[3] Stark T, Livas L, Kyprianou N. Inflammation in prostate cancer progression and therapeutic targeting. Transl. Androl. Urol. 2015;4(4):455–63. doi: 10.3978/j.issn.2223-4683.2015.04.12 26816843 PMC4708587

[4] National Cancer Insitute. Cancer Stat Facts: Cancer of Any Site. Natl. Cancer Inst. 2018. [Cited 2020 March 17]. Available from: https://www.seer.cancer.gov

[5] Debes JD, Tindall DJ. Mechanisms of androgen-refractory prostate cancer. N. Engl. J. Med. 2004;351(15):1488–90. doi: 10.1056/NEJMp048178 15470210

[6] American Cancer Society. Facts & Figures 2019. Am. Cancer Soc. 2019. [Cited 2020 March 17]. Available from: https://www.cancer.org

[7] Lee JW, Bae SH, Jeong JW, Kim SH, Kim KW. Hypoxia-inducible factor (HIF-1)α: Its protein stability and biological functions. Exp. Mol. Med. 2004;36(1):1–12. doi: 10.1038/emm.2004.1 15031665

[8] Forbes SA, Bindal N, Bamford S, Cole C, Kok CY, Beare D, et al. COSMIC: Mining complete cancer genomes in the catalogue of somatic mutations in cancer. Nucleic Acids Res. 2011;39:D945–D950. doi: 10.1093/nar/gkq929 20952405 PMC3013785

[9] Kalluri R, Weinberg RA. The basics of epithelial-mesenchymal transition. J. Clin. Invest. 2009;119(6):1420–8. doi: 10.1172/JCI39104 19487818 PMC2689101

[10] Vu T, Datta PK. Regulation of EMT in colorectal cancer: A culprit in metastasis. Cancers (Basel). 2017;9(12):171. 10.3390/cancers9120171 PMC5742819 29258163

[11] Mylavarapu S, Kumar H, Kumari S, Sravanthi LS, Jain M, Basu A, et al. Activation of Epithelial-Mesenchymal Transition and Altered β-Catenin Signaling in a Novel Indian Colorectal Carcinoma Cell Line. Front Oncol. 2019;9:54. doi: 10.3389/fonc.2019.00054 30828563 PMC6385509

[12] Mani SA, Guo W, Liao MJ, Eaton EN, Ayyanan A, Zhou AY, et al. The Epithelial-Mesenchymal Transition Generates Cells with Properties of Stem Cells. Cell. 2008;133(4):704–715. doi: 10.1016/j.cell.2008.03.027 18485877 PMC2728032

[13] Reya T, Morrison SJ, Clarke MF, Weissman IL. Stem cells, cancer, and cancer stem cells. Nature. 2001;414(6859):105–11. doi: 10.1038/35102167 11689955

[14] Valenta T, Hausmann G, Basler K. The many faces and functions of Î 2-catenin. EMBO J. 2012; 31(12):2714–36. doi: 10.1038/emboj.2012.150 22617422 PMC3380220

[15] Weis WI, Nelson WJ. Re-solving the cadherin-catenin-actin conundrum. J. Biol. Chem. 2006;281(47):35593–7. doi: 10.1074/jbc.R600027200 17005550 PMC3368706

[16] Ben-Ze’ev A, Basu S, Haase G. Wnt signaling in cancer stem cells and colon cancer metastasis. F1000Research. 2016;5:F1000. doi: 10.12688/f1000research.7579.1 27134739 PMC4841194

[17] Hinck L, Näthke IS, Papkoff J, Nelson WJ. Dynamics of cadherin/catenin complex formation: Novel protein interactions and pathways of complex assembly. J Cell Biol. 1994;125(6):1327–40. doi: 10.1083/jcb.125.6.1327 8207061 PMC2290923

[18] Huber AH, Weis WI. The structure of the β-catenin/E-cadherin complex and the molecular basis of diverse ligand recognition by β-catenin. Cell. 2001;105(3):391–402. doi: 10.1016/s0092-8674(01)00330-0 11348595

[19] Grant CM, Kyprianou N. Epithelial mesenchymal transition (EMT) in prostate growth and tumor progression. Transl. Androl. Urol. 2013;2(3):202–211. doi: 10.3978/j.issn.2223-4683.2013.09.04 25346895 PMC4208065

[20] Qin Y, Zhao W, Chai D, Ou Y, Wu S, Wu Q, et al. Clinicopathological significance of SOX4, β-catenin and TCF4 expression in esophageal squamous cell carcinoma. 2019;12(8):10559–68.

[21] Aberle H, Bauer A, Stappert J, Kispert A, Kemler R. β-catenin is a target for the ubiquitin-proteasome pathway. EMBO J. 1997;16(13):3797–804. doi: 10.1093/emboj/16.13.3797 9233789 PMC1170003

[22] Schuijers J, Mokry M, Hatzis P, Cuppen E, Clevers H. Wnt-induced transcriptional activation is exclusively mediated by TCF/LEF. EMBO J. 2014;33(2):146–56. doi: 10.1002/embj.201385358 24413017 PMC3989608

[23] Korinek V, Barker N, Morin PJ, Van Wichen D, De Weger R, Kinzler KW, et al. Constitutive transcriptional activation by a β-catenin-Tcf complex in APC(-/-) colon carcinoma. Science (80-). 1997;275(5307):1784–7. doi: 10.1126/science.275.5307.1784 9065401

[24] Lickert H, Domon C, Huls G, Wehrle C, Duluc I, Clevers H, et al. Wnt/β-catenin signaling regulates the expression of the homeobox gene Cdx1 in embryonic intestine. Development. 2000;127(17):3805–13. 10934025 10.1242/dev.127.17.3805

[25] Sinner D, Kordich JJ, Spence JR, Opoka R, Rankin S, Lin S-CJ, et al. Sox17 and Sox4 Differentially Regulate -Catenin/T-Cell Factor Activity and Proliferation of Colon Carcinoma Cells. Mol Cell Biol. 2007;27(22):7802–15. doi: 10.1128/MCB.02179-06 17875931 PMC2169141

[26] Schepers GE, Teasdale RD, Koopman P. Twenty pairs of Sox: Extent, homology, and nomenclature of the mouse and human Sox transcription factor gene families. Dev. Cell. 2002; 3(2):167–70. doi: 10.1016/s1534-5807(02)00223-x 12194848

[27] Schilham MW, Oosterwegel MA, Moerer P, Ya J, De Boert PAJ, Van De Wetering M, et al. Defects in cardiac outflow tract formation and pro-B-lymphocyte expansion in mice lacking Sox-4. Nature. 1996;380(6576):711–4. doi: 10.1038/380711a0 8614465

[28] Dy P, Penzo-Méndez A, Wang H, Pedraza CE, Macklin WB, Lefebvre V. The three SoxC proteins—Sox4, Sox11 and Sox12—Exhibit overlapping expression patterns and molecular properties. Nucleic Acids Res. 2008;36(9):3101–17. doi: 10.1093/nar/gkn162 18403418 PMC2396431

[29] Vervoort SJ, Van Boxtel R, Coffer PJ. The role of SRY-related HMG box transcription factor 4 (SOX4) in tumorigenesis and metastasis: Friend or foe¿. Oncogene. 2013;32(29):3397–409. doi: 10.1038/onc.2012.506 23246969

[30] Bergsland M, Ramsköld D, Zaouter C, Klum S, Sandberg R, Muhr J. Sequentially acting Sox transcription factors in neural lineage development. Genes Dev. 2011;25(23):2453–64. doi: 10.1101/gad.176008.111 22085726 PMC3243056

[31] Shi S, Cao X, Gu M, You B, Shan Y, You Y. Upregulated Expression of SOX4 Is Associated with Tumor Growth and Metastasis in Nasopharyngeal Carcinoma. Dis Markers. 2015;2015:658141. doi: 10.1155/2015/658141 26578818 PMC4633550

[32] Moreno CS. The sex-determining region Y-Box 4 and homeobox C6 transcriptional networks in prostate cancer progression: Crosstalk with the Wnt, Notch, and PI3K pathways. Am J Pathol. 2010;176(2):518–27. doi: 10.2353/ajpath.2010.090657 20019190 PMC2808058

[33] Graham JD, Hunt SMN, Tran N, Clarke CL. Regulation of the expression and activity by progestins of a member of the SOX gene family of transcriptional modulators. J Mol Endocrinol. 1999;22(3):295–304. doi: 10.1677/jme.0.0220295 10343288

[34] Castillo SD, Matheu A, Mariani N, Carretero J, Lopez-Rios F, Lovell-Badge R, et al. Novel transcriptional targets of the SRY-HMG box transcription factor SOX4 link its expression to the development of small cell lung cancer. Cancer Res. 2012;72(1):176–86. doi: 10.1158/0008-5472.CAN-11-3506 22084397

[35] Fang CL, Hseu YC, Lin YF, Hung ST, Tai C, Uen YH, et al. Clinical and Prognostic Association of Transcription Factor SOX4 in Gastric Cancer. PLoS One. 2012;7(12):e52804. doi: 10.1371/journal.pone.0052804 23285187 PMC3527618

[36] Jafarnejad SM, Wani AA, Martinka M, Li G. Prognostic significance of Sox4 expression in human cutaneous melanoma and its role in cell migration and invasion. Am J Pathol. 2010;177(6):2741–52. doi: 10.2353/ajpath.2010.100377 20952589 PMC2993272

[37] Aaboe M, Birkenkamp-Demtroder K, Wiuf C, Sørensen FB, Thykjaer T, Sauter G, et al. SOX4 expression in bladder carcinoma: Clinical aspects and in vitro functional characterization. Cancer Res. 2006;66(7):3434–42. doi: 10.1158/0008-5472.CAN-05-3456 16585165

[38] Bucala R, Donnelly SC. Macrophage Migration Inhibitory Factor: A Probable Link between Inflammation and Cancer. Immunity. 2007;26(3):281–5. doi: 10.1016/j.immuni.2007.03.005 17376392

[39] Funamizu N, Hu C, Lacy C, Schetter A, Zhang G, He P, et al. Macrophage migration inhibitory factor induces epithelial to mesenchymal transition, enhances tumor aggressiveness and predicts clinical outcome in resected pancreatic ductal adenocarcinoma. Int J Cancer. 2012;132(4):785–94. 10.1002/ijc.27736 PMC3488363 22821831

[40] Calandra T, Roger T. Macrophage migration inhibitory factor: A regulator of innate immunity. Nat. Rev. Immunol. 2003;3(10):791–800. doi: 10.1038/nri1200 14502271 PMC7097468

[41] Santos LL, Morand EF. Macrophage migration inhibitory factor: A key cytokine in RA, SLE and atherosclerosis. Clin. Chim. Acta. 2009;399(1–2):1–7. doi: 10.1016/j.cca.2008.09.014 18838066

[42] Sánchez-Zamora YI, Rodriguez-Sosa M. The role of MIF in type 1 and type 2 diabetes mellitus. J. Diabetes Res. 2014;2014:804519. doi: 10.1155/2014/804519 24527464 PMC3910331

[43] Verjans E, Noetzel E, Bektas N, Schütz AK, Lue H, Lennartz B, et al. Dual role of macrophage migration inhibitory factor (MIF) in human breast cancer. BMC Cancer. 2009;9:230. doi: 10.1186/1471-2407-9-230 19602265 PMC2716369

[44] Mawhinney L, Armstrong ME, O’ Reilly C, Bucala R, Leng L, Fingerle-Rowson G, et al. Macrophage migration inhibitory factor (MIF) enzymatic activity and lung cancer. Mol Med. 2014;20(1):729–35. 10.2119/molmed.2014.00136 PMC4398672 25826675

[45] Bando H, Matsumoto G, Bando M, Muta M, Ogawa T, Funata N, et al. Expression of macrophage migration inhibitory factor in human breast cancer: Association with nodal spread. Japanese J Cancer Res. 2002;93(4):389–96. doi: 10.1111/j.1349-7006.2002.tb01269.x 11985788 PMC5927007

[46] Gordon-Weeks AN, Lim SY, Yuzhalin AE, Jones K, Muschel R. Macrophage migration inhibitory factor: A key cytokine and therapeutic target in colon cancer. Cytokine Growth Factor Rev. 2015;26(4):451–61. doi: 10.1016/j.cytogfr.2015.03.002 25882738

[47] Mamoori A, Wahab R, Vider J, Gopalan V, Lam AK yin. The tumour suppressor effects and regulation of cancer stem cells by macrophage migration inhibitory factor targeted miR-451 in colon cancer. Gene. 2019;697:165–174. doi: 10.1016/j.gene.2019.02.046 30802541

[48] Soumoy L, Kindt N, Ghanem G, Saussez S, Journe F. Role of macrophage migration inhibitory factor (Mif) in melanoma. Cancers (Basel). 2019;11(4):529. 10.3390/cancers11040529 PMC6520935 31013837

[49] Presti M, Mazzon E, Basile MS, Petralia MC, Bramanti A, Colletti G, et al. Overexpression of macrophage migration inhibitory factor and functionally-related genes, D-DT, CD74, CD44, CXCR2 and CXCR4, in glioblastoma. Oncol Lett. 2018;16(3):2881–2886. doi: 10.3892/ol.2018.8990 30127875 PMC6096183

[50] Kamimura A, Kamachi M, Nishihira J, Ogura S, Isobe H, Dosaka-Akita H, et al. Intracellular distribution of macrophage migration inhibitory factor predicts the prognosis of patients adenocarcinoma of the lung. Cancer. 2000;89(2):334–41. 10918163

[51] Penticuff JC, Woolbright BL, Sielecki TM, Weir SJ, Taylor JA. MIF family proteins in genitourinary cancer: tumorigenic roles and therapeutic potential. Nat. Rev. Urol. 2019;16(5):318–328. doi: 10.1038/s41585-019-0171-9 30914802

[52] Choudhary S, Hegde P, Pruitt JR, Sielecki TM, Choudhary D, Scarpato K, et al. Macrophage migratory inhibitory factor promotes bladder cancer progression via increasing proliferation and angiogenesis. Carcinogenesis. 2013;34(12):2891–9. doi: 10.1093/carcin/bgt239 23825153 PMC3845890

[53] Cheng SP, Liu CL, Chen MJ, Chien MN, Leung CH, Lin CH, et al. CD74 expression and its therapeutic potential in thyroid carcinoma. Endocr Relat Cancer. 2015;22(2):179–90. doi: 10.1530/ERC-14-0269 25600560

[54] Hussain F, Freissmuth M, Völkel D, Thiele M, Douillard P, Antoine G, et al. Human anti-macrophage migration inhibitory factor antibodies inhibit growth of human prostate cancer cells in vitro and in vivo. Mol Cancer Ther. 2013;12(7):1223–34. doi: 10.1158/1535-7163.MCT-12-0988 23619302

[55] Amin MB. AJCC Cancer Staging System, 8th Edition. Am Jt Commitee Cancer. 2017;67(2):93–99. 10.3322/caac.21388 28094848

[56] Thul PJ, Lindskog C. The human protein atlas: A spatial map of the human proteome. Protein Sci. 2018;27(1):233–244. doi: 10.1002/pro.3307 28940711 PMC5734309

[57] Saha B, Arase A, Imam SS, Tsao-Wei D, Naritoku WY, Groshen S, et al. Overexpression of E-cadherin and β-catenin proteins in metastatic prostate cancer cells in bone. Prostate. 2008;68(1):78–84. doi: 10.1002/pros.20670 18008331

[58] Putzke AP, Ventura AP, Bailey AM, Akture C, Opoku-Ansah J, Çeliktaş M, et al. Metastatic progression of prostate cancer and E-cadherin: Regulation by ZEB1 and Src family kinases. Am J Pathol. 2011;179(1):400–10. doi: 10.1016/j.ajpath.2011.03.028 21703419 PMC3123858

[59] Sánchez-Tilló E, Lázaro A, Torrent R, Cuatrecasas M, Vaquero EC, Castells A, et al. ZEB1 represses E-cadherin and induces an EMT by recruiting the SWI/SNF chromatin-remodeling protein BRG1. Oncogene. 2010;29(24):3490–500. doi: 10.1038/onc.2010.102 20418909

[60] Nakamura M, Tokura Y. Epithelial-mesenchymal transition in the skin. J. Dermatol. Sci. 2011;61(1):7–13. doi: 10.1016/j.jdermsci.2010.11.015 21167690

[61] Rubin MA, Mucci NR, Figurski J, Fecko A, Pienta KJ, Day ML. E-cadherin expression in prostate cancer: A broad survey using high-density tissue microarray technology. Hum Pathol. 2001;32(7):690–7. doi: 10.1053/hupa.2001.25902 11486167

[62] Mao Q, Zheng X, Yang K, Qin J, Bai Y, Jia X, et al. Suppression of migration and invasion of PC3 prostate cancer cell line via activating E-cadherin expression by small activating RNA. Cancer Invest. 2010;28(10):1013–8. doi: 10.3109/07357900802620844 20690797

[63] Isaacs WB, Carter BS, Debruyne FMJ, Schalken JA. Expression of the Cellular Adhesion Molecule E-Cadherin Is Reduced or Absent in High-Grade Prostate Cancer. Cancer Res. 1992;52(18):5104–9. 1516067

[64] Jaggi M, Johansson SL, Baker JJ, Smith LM, Galich A, Balaji KC. Aberrant expression of E-cadherin and beta-catenin in human prostate cancer. Urol Oncol Semin Orig Investig. 2005;23(6):402–6. doi: 10.1016/j.urolonc.2005.03.024 16301117

[65] Price N, Reddy GK, Peck S, Jain VK. 40th Annual Meeting of the American Society of Clinical Oncology, New Orleans, Louisiana. Clin Lymphoma. 2004. Jun 5–8, p. 9570.

[66] Scharer CD, McCabe CD, Ali-Seyed M, Berger MF, Bulyk ML, Moreno CS. Genome-wide promoter analysis of the SOX4 transcriptional network in prostate cancer cells. Cancer Res. 2009;69(2):709–17. doi: 10.1158/0008-5472.CAN-08-3415 19147588 PMC2629396

[67] Liu Y, Zeng S, Jiang X, Lai D, Su Z. SOX4 induces tumor invasion by targeting EMT-Related pathway in prostate cancer. Tumor Biol. 2017;39(5):1010428317694539. doi: 10.1177/1010428317694539 28466783

[68] Bilir B, Osunkoya AO, Wiles WG, Sannigrahi S, Lefebvre V, Metzger D, et al. SOX4 is essential for prostate tumorigenesis initiated by PTEN ablation. Cancer Res. 2016;76(5):1112–21. doi: 10.1158/0008-5472.CAN-15-1868 26701805 PMC4779598

[69] Saegusa M, Hashimura M, Kuwata T. Sox4 functions as a positive regulator of β-catenin signaling through upregulation of TCF4 during morular differentiation of endometrial carcinomas. Lab Investig. 2012;92(4):511–21. doi: 10.1038/labinvest.2011.196 22231735

[70] Wang L, Zhang J, Yang X, Chang YWY, Qi M, Zhou Z, et al. SOX4 is associated with poor prognosis in prostate cancer and promotes epithelial-mesenchymal transition in vitro. Prostate Cancer Prostatic Dis. 2013;16(4):301–7. doi: 10.1038/pcan.2013.25 23917306

[71] Lin CM, Fang CL, Hseu YC, Chen CL, Wang JW, Hsu SL, et al. Clinical and Prognostic Implications of Transcription Factor SOX4 in Patients with Colon Cancer. PLoS One. 2013;8(6):e67128. doi: 10.1371/journal.pone.0067128 23826209 PMC3694951

[72] Liu P, Ramachandran S, Seyed MA, Scharer CD, Laycock N, Dalton WB, et al. Sex-determining region Y box 4 is a transforming oncogene in human prostate cancer cells. Cancer Res. 2006;66(8):4011–9. doi: 10.1158/0008-5472.CAN-05-3055 16618720

[73] De Marzo AM, DeWeese TL, Platz EA, Meeker AK, Nakayama M, Epstein JI, et al. Pathological and molecular mechanisms of prostate carcinogenesis: Implications for diagnosis, detection, prevention, and treatment. J. Cell. Biochem. 2004;91(3):459–77. doi: 10.1002/jcb.10747 14755677

[74] Guo F, Fu X, Yang J, Zhang X, Liu D, Feng W, et al. Role of macrophage migration inhibitory factor in mesenchymal epithelial transition of cervical carcinoma cells. Int J Clin Exp Pathol. 2017;13(11):2916. 33284881 PMC7716129

[75] Roger T, David J, Glauser MP, Calandra T. MIF regulates innate immune responses through modulation of Toll-like receptor 4. Nature. 2001;414(6866):920–4. doi: 10.1038/414920a 11780066

[76] Meyer-Siegler K, Hudson PB. Enhanced expression of macrophage migration inhibitory factor in prostatic adenocarcinoma metastases. Urology. 1996;48(3):448–52. doi: 10.1016/S0090-4295(96)00207-5 8804500

[77] Ohkawara T, Nishihira J, Takeda H, Asaka M, Sugiyama T. Pathophysiological roles of macrophage migration inhibitory factor in gastrointestinal, hepatic, and pancreatic disorders. J. Gastroenterol. 2005;40(2):117–22. doi: 10.1007/s00535-004-1526-3 15770393

[78] Meyer-Siegler KL, Bellino MA, Tannenbaum M. Macrophage migration inhibitory factor evaluation compared with prostate specific antigen as a biomarker in patients with prostate carcinoma. Cancer. 2002;94(5):1449–56. doi: 10.1002/cncr.10354 11920501

[79] Guda MR, Rashid MA, Asuthkar S, Jalasutram A, Caniglia JL, Tsung AJ, et al. Pleiotropic role of macrophage migration inhibitory factor in cancer. Am J Cancer Res. 2019;9(12):2760–2773. 31911860 PMC6943360

[80] Ren Y, Tsui HT, Poon RTP, Ng IOL, Li Z, Chen Y, et al. Macrophage migration inhibitory factor: Roles in regulating tumor cell migration and expression of angiogenic factors in hepatocellular carcinoma. Int J Cancer. 2003;107(1):22–9. doi: 10.1002/ijc.11287 12925952

[81] Charan M, Das S, Mishra S, Chatterjee N, Varikuti S, Kaul K, et al. Macrophage migration inhibitory factor inhibition as a novel therapeutic approach against triple-negative breast cancer. Cell Death Dis. 2020;11(9):774. doi: 10.1038/s41419-020-02992-y 32943608 PMC7498597

[82] del Vecchio MT, Tripodi SA, Arcuri F, Pergola L, Hako L, Vatti R, et al. Macrophage migration inhibitory factor in prostatic adenocarcinoma: Correlation with tumor grading and combination endocrine treatment-related changes. Prostate. 2000;45(1):51–7. doi: 10.1002/1097-0045(20000915)45:1&lt;51::aid-pros6&gt;3.0.co;2-9 10960842

[83] Chen YC, Zhang XW, Niu XH, Xin DQ, Zhao WP, Na YQ, et al. Macrophage migration inhibitory factor is a direct target of HBP1-mediated transcriptional repression that is overexpressed in prostate cancer. Oncogene. 2010;29(21):3067–78. doi: 10.1038/onc.2010.97 20383199

[84] Pei XJ, Wu TT, Li B, Tian XY, Li Z, Yang QX. Increased expression of macrophage migration inhibitory factor and DJ-1 contribute to cell invasion and metastasis of nasopharyngeal carcinoma. Int J Med Sci. 2013;11(1):106–15. doi: 10.7150/ijms.7264 24396292 PMC3880997

[85] Yao Y, Deng Q, Song W, Zhang H, Li Y, Yang Y, et al. MIF Plays a Key Role in Regulating Tissue-Specific Chondro-Osteogenic Differentiation Fate of Human Cartilage Endplate Stem Cells under Hypoxia. Stem Cell Reports. 2016;7(2):249–62. doi: 10.1016/j.stemcr.2016.07.003 27509135 PMC4982989

[86] Ohta S, Misawa A, Lefebvre V, Okano H, Kawakami Y, Toda M. Sox6 Up-Regulation by Macrophage Migration Inhibitory Factor Promotes Survival and Maintenance of Mouse Neural Stem/Progenitor Cells. PLoS One. 2013;8(9):e74315. doi: 10.1371/journal.pone.0074315 24066135 PMC3774630

