# Supplementary material for: Clinicopathological significance of the EMT-related proteins and their interrelationships in prostate cancer. An immunohistochemical study
Source: PLoS One. 2021 Jun 22;16(6):e0253112. doi: 10.1371/journal.pone.0253112 (PMC8219170; doi:10.1371/journal.pone.0253112)
Supplement: S1 Table — (DOCX) [file pone.0253112.s001.docx]

S1 Table. The additional values of significant correlations between protein expression and clinicopathological features.

| **Correlation between protein expression and clinicopathological features** | | **Mean** | **Median** | **Min** | **Max** | **25^th^ percentil** | **75^th^ percentil** | **SD** | **P value** | **Figure** |
| --- | --- | --- | --- | --- | --- | --- | --- | --- | --- | --- |
| General expression of β-catenin and lymph node metastasis | N0 | 10.6 | 12.0 | 4.0 | 12.0 | 8.0 | 12.0 | 2.1 | 0.0004 | Fig A |
|  | N1 | 7.6 | 8.0 | 3.0 | 4.0 | 6.0 | 9.0 | 3.0 |  |  |
| General expression of β-catenin and angioinvasion | (-) | 10.4 | 12.0 | 3.0 | 12.0 | 8.0 | 12.0 | 2.3 | 0.012 | Fig B |
|  | (+) | 8.0 | 8.0 | 4.0 | 12.0 | 6.0 | 12.0 | 3.2 |  |  |
| General expression of β-catenin and the plugs of cancer cells in the vessel | (-) | 10.4 | 12.0 | 3.0 | 12.0 | 8.0 | 12.0 | 2.3 | 0.027 | Fig C |
|  | (+) | 8.3 | 8.0 | 4.0 | 12.0 | 6.0 | 12.0 | 3.2 |  |  |
| Membrane expression of β-catenin and distant metastasis | M0 | 7.1 | 6.0 | 2.0 | 12.0 | 6.0 | 9.0 | 2.4 | 0.021 | Fig D |
|  | M1 | 4.9 | 6.0 | 1.0 | 9.0 | 4.0 | 6.0 | 2.4 |  |  |
| Membrane expression of β-catenin and prostate capsule infiltration on the left side | (-) | 7.3 | 6.0 | 2.0 | 12.0 | 6.0 | 9.0 | 2.8 | 0.028 | Fig E |
|  | (+) | 6.4 | 6.0 | 1.0 | 9.0 | 6.0 | 8.0 | 2.1 |  |  |
| Membrane expression of E-cadherin and both sides infiltration through prostate capsule | (-) | 7.8 | 9.0 | 2.0 | 12.0 | 6.0 | 9.0 | 2.6 | 0.027 | Fig F |
|  | (+) | 8.1 | 9.0 | 4.0 | 12.0 | 6.0 | 9.0 | 2.1 |  |  |
| Nuclear expression of MIF and Gleason score | GS6 | 2.6 | 0.0 | 0.0 | 9.0 | 0.0 | 6.0 | 3.6 | 0.039 | Fig G |
|  | GS7 | 3.6 | 4.0 | 0.0 | 12.0 | 0.0 | 6.0 | 3.6 |  |  |
|  | GS8 | 7.3 | 6.0 | 3.0 | 12.0 | 4.0 | 12.0 | 3.8 |  |  |
|  | GS9 | 5.1 | 6.0 | 0.0 | 12.0 | 0.0 | 9.0 | 4.3 |  |  |
| Nuclear expression of MIF and lymph node metastasis | N0 | 3.6 | 3.5 | 0.0 | 12.0 | 0.0 | 6.0 | 3.6 | 0.003 | Fig H |
|  | N1 | 7.0 | 6.0 | 0.0 | 12.0 | 6.0 | 12.0 | 4.4 |  |  |
