# Supplementary material for: Clinicopathological significance of the EMT-related proteins and their interrelationships in prostate cancer. An immunohistochemical study
Source: PLoS One. 2021 Jun 22;16(6):e0253112. doi: 10.1371/journal.pone.0253112 (PMC8219170; doi:10.1371/journal.pone.0253112)
Supplement: S2 Table — (DOCX) [file pone.0253112.s002.docx]

S2 Table. Comparison of significant values for Mann-Whitney U test.

| **Positive protein expression** | **Clinicopathological features** | |
| --- | --- | --- |
| nuclear β-catenin | N status | |
|  | **Z** | **P** |
|  | -3.9 | 0.0004 |
| nuclear MIF | N status | |
|  | **Z** | **P** |
|  | 2.9 | 0.0031 |
| membrane β-catenin | M status | |
|  | **Z** | **P** |
|  | -2.4 | 0.021 |
| general β-catenin | Angioinvasion | |
|  | **Z** | **P** |
|  | -2.8 | 0.012 |
| membrane β-catenin | Left side invaded area of the prostate capsule | |
|  | **Z** | **P** |
|  | -2.3 | 0.028 |
| membrane E-cadherin | Both sides invaded area of the prostate capsule | |
|  | **Z** | **P** |
|  | 2.3 | 0.027 |
| cytoplasmic MIF | Both sides invaded area of the prostate capsule | |
|  | **Z** | **P** |
|  | -2.5 | 0.022 |
| general β-catenin | Plugs of cancer cells in the blood vessels | |
|  | **Z** | **P** |
|  | -2.5 | 0.027 |
